# Changes in both trans- and cis-regulatory elements mediate insecticide resistance in a lepidopteron pest, *Spodoptera exigua*

**DOI:** 10.1371/journal.pgen.1009403

**Published:** 2021-03-09

**Authors:** Bo Hu, He Huang, Songzhu Hu, Miaomiao Ren, Qi Wei, Xiangrui Tian, Mohammed Esmail Abdalla Elzaki, Chris Bass, Jianya Su, Subba Reddy Palli

**Affiliations:** 1 Key Laboratory of Integrated Management of Crop Diseases and Pests (Ministry of Education), College of Plant Protection, Nanjing Agricultural University, Nanjing, China; 2 College of Agriculture, Fujian Agriculture and Forestry University, Fuzhou, China; 3 College of Life and Environmental Sciences, Biosciences, University of Exeter, Penryn Campus, Penryn, United Kingdom; 4 Department of Entomology, University of Kentucky, Lexington, Kentucky, United States of America; Universidad de Valparaiso, CHILE

## Abstract

The evolution of insect resistance to insecticides is frequently associated with overexpression of one or more cytochrome P450 enzyme genes. Although overexpression of CYP450 genes is a well-known mechanism of insecticide resistance, the underlying regulatory mechanisms are poorly understood. Here we uncovered the mechanisms of overexpression of the P450 gene, *CYP321A8* in a major pest insect, *Spodoptera exigua* that is resistant to multiple insecticides. *CYP321A8* confers resistance to organophosphate (chlorpyrifos) and pyrethroid (cypermethrin and deltamethrin) insecticides in this insect. Constitutive upregulation of transcription factors *CncC/Maf* are partially responsible for upregulated expression of *CYP321A8* in the resistant strain. Reporter gene assays and site-directed mutagenesis analyses demonstrated that *CncC/Maf* enhanced the expression of *CYP321A8* by binding to specific sites in the promoter. Additional *cis*-regulatory elements resulting from a mutation in the *CYP321A8* promoter in the resistant strain facilitates the binding of the orphan nuclear receptor, *Knirps*, and enhances the promoter activity. These results demonstrate that two independent mechanisms; overexpression of transcription factors and mutations in the promoter region resulting in a new *cis*-regulatory element that facilitates binding of the orphan nuclear receptor are involved in overexpression of *CYP321A8* in insecticide-resistant *S*. *exigua*.

## Author summary

Insect pests developing resistance to insecticides used for their control is a major problem in agriculture. Many pests including the beet armyworm, *Spodoptera exigua* have developed resistance to insecticides used for their control. Information on the mechanisms of resistance would help in resistance management programs. Overexpression of detoxifying enzymes were associated with insecticide resistance, but their functions and regulatory mechanisms are still unidentified. The expression levels of P450 genes between susceptible and resistant strains of *S*. *exigua* were compared and *CYP321A8* was identified as the major contributor to resistance to organophosphate and pyrethroid insecticides. Further studies uncovered two independent but synergistic mechanisms; constitutive upregulation of b-Zip transcription factors and mutations in the promoter that facilitates the binding of an orphan nuclear receptor, *Knirps* contributing to increase in the expression of *CYP321A8* and resistance to multiple insecticides in *S*. *exigua*.

## Introduction

Insects often develop resistance to insecticides that are used for their control. This microevolutionary change in response to environmental challenges constitutes a constant battle between insects and humans [1–3]. Insects develop resistance to insecticides through multiple mechanisms; enhanced insecticide detoxification (metabolic resistance) and target-site insensitivity (target-site resistance) are among the major mechanisms. Cytochrome P450 (P450) enzymes that are capable of metabolizing synthetic insecticides and plant compounds including toxins play a major role in metabolic resistance [4]. Overexpression of genes coding for P450s has been shown to associate with resistance to insecticides in a wide range of insect species [5–8]. For example, overexpression of *CYP6G1* confers resistance to the insecticides DDT and imidacloprid in *Drosophila melanogaster* [9].

Mutations in *cis*-regulatory elements in the promoter regions of P450 genes and/or changes in the expression level of transcription factors binding to these *cis*-regulatory elements may contribute to enhanced expression of P450 genes [10–14]. Mutations in *cis*-acting elements in P450 promoters have been shown to cause constitutive overexpression of P450 genes [2,15–18]. In *D*. *melanogaster*, upregulation of *CYP6G1* gene results from a TE insertion in the promoter sequence [3,19]. Members of nuclear receptors (NRs), basic-leucine zipper (bZIP) and basic-helix-loop-helix/per-ARNT-SIM (bHLH-PAS) superfamilies are known to mediate insecticide resistance [20]. In arthropods, constitutive overexpression of nuclear receptors/transcription factors belonging to these superfamilies contribute to increasing the expression of P450s responsible for metabolic resistance. Increase in the expression of the nuclear receptor, *FTZ-F1* in *Plutella xylostella* causes overexpression of *CYP6BG1* that metabolizes chlorantraniliprole [21]. Similarly, the Aryl hydrocarbon receptor (AhR) belonging to the bHLH-PAS protein family [20] regulates *CYP6DA2* in *Aphis gossypii* conferring gossypol and spirotetramat tolerance [22]. The heterodimer bZIP transcription factors *Nrf2* and *Maf* play a significant role in regulation of detoxification genes associated with oxidative or xenobiotic response in humans [23]. *CncC*, the insect ortholog of *Nrf2* along with its heterodimer partner, *Maf-S* also regulate the expression of detoxification genes associated with metabolism of xenobiotics including insecticides and plant chemicals [24,25]. For example, these transcription factors control the overexpression of P450 genes in *D*. *melanogaster* [26] that are resistant to insecticides. Despite growing understanding of the role of changes in *cis*-acting and *trans*-acting elements in the regulation of P450 resistance genes, the relative importance of the two mechanisms and whether they primarily act in isolation or in combination remains poorly understood.

*Spodoptera exigua* is a worldwide pest that causes serious damage to many crops [27], and has evolved resistance to 38 insecticides [28]. This work was conducted to investigate the molecular basis of resistance of this species to organophosphate and pyrethroid insecticides. We found that a combination of *cis*- and *trans*-acting factors act in tandem to regulate a cytochrome P450 leading to resistance to multiple insecticides.

## Results

### Insecticide resistance is connected with overexpression of the P450, *CYP321A8*

**Fig 1:**
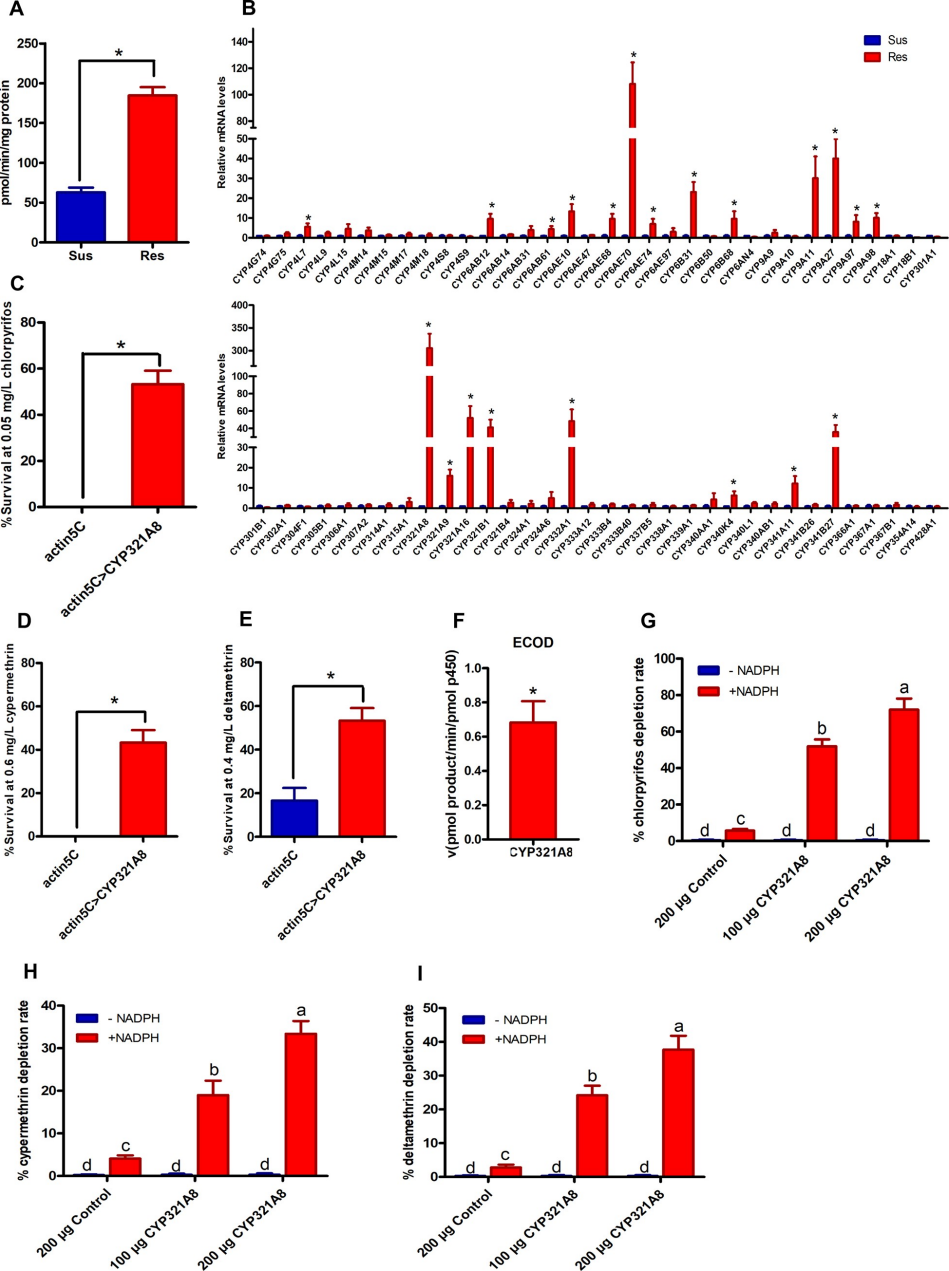
Overexpression of *CYP321A8* confers resistance to multiple insecticides in *S*. *exigua*. (A) P450 activity in the larvae of the susceptible and resistant strains of *S*. *exigua*. P450 monooxygenase activity was evaluated by measuring ethoxycoumarin-O-deethylase (ECOD) activity. A significant difference in enzymatic activities is indicated using an asterisk (Student’s t-test, p < 0.05). Error bars indicate SD. (B) Relative expression of P450 genes in the susceptible and resistant strains of *S*. *exigua* as determined by RT-qPCR. Error bars display SD. A significant difference in expression between the susceptible and resistant strains is indicated using an asterisk (ANOVA with post-hoc Tukey’s HSD, p < 0.05). The sensitivity of transgenic *D*. *melanogaster* to chlorpyrifos (C), cypermethrin (D) and deltamethrin (E). Error bars display SD. Significant differences in mortality between lines expressing CYP321A8 and control flies without the transgene are indicated using an asterisk (Student’s t-test, p < 0.05). (F) Metabolic activity of recombinant CYP321A8 enzyme against a model fluorescent substrate. The rate of O-dealkylation of 7-ethoxy coumarin (ECOD) is shown. Error bars display SD. Metabolism of chlorpyrifos (G), cypermethrin (H) and deltamethrin (I) metabolism by recombinant CYP321A8. Error bars display SD values. Significant differences (p < 0.05) in metabolism are denoted using letters above bars (ANOVA with post-hoc Tukey’s HSD).

In order to study the relationship between CYP450 and insecticide resistance in *S*. *exigua*, bioassay and enzyme activity analysis were performed. Compared with the susceptible strain, the resistant strain displayed 935, 563 and 305-fold resistance to chlorpyrifos, cypermethrin, and deltamethrin (S1 Table). Bioassays revealed that the P450 inhibitor, piperonyl butoxide (PBO) significantly enhanced the toxicity of chlorpyrifos, cypermethrin, and deltamethrin in the resistant strain (synergistic ratios of 8.1, 3.6 and 3.0 respectively) (S1 Table). In contrast, PBO showed no synergistic effect in the susceptible strain. Biochemical measurement of P450 activity using the model fluorescent substrate 7-ethoxycoumarin revealed a significantly higher P450 activity in the resistant strain (2.93-fold, p-value < 0.05) compared with the susceptible strain (Fig 1A). These results suggest that the resistance to chlorpyrifos, cypermethrin and deltamethrin may be due to enhanced P450 activity in the resistant strain. Therefore, we will focus on the relationship between CYP450 and insecticide resistance in this study, and GSTs and ESTs will be reported separately.

To identify P450s involved in resistance, we analyzed the expression level of 68 P450s between the resistant and susceptible strains (Fig 1B). Twenty-one of these (30% of genes analyzed) showed more than 2-fold up-regulation (p-value < 0.05) in the resistant strain suggesting multiple P450s may contribute to insecticide resistance in *S*. *exigua*. However, among these CYP321A8 was particularly highly expressed in the resistant strain (306-fold higher). For this reason, subsequent studies on the molecular mechanisms responsible for P450 gene overexpression in the resistant strain focused on *CYP321A8*.

### Overexpression of *CYP321A8* confers resistance to insecticides

To determine whether the upregulation of *CYP321A8* is sufficient to confer insecticide resistance, the Act5C-GAL4 strain and GAL4/UAS system were used for expression of *CYP321A8* (Act5C-CYP321A8) in transgenic *D*. *melanogaster*. RT-PCR (S1A Fig) and RT-qPCR (S1B Fig) were used to confirm the expression level of *CYP321A8* in F_1_ progeny. When insects expressing CYP321A8 were treated with 0.05 ppm chlorpyrifos for 3 days, 47% mortality was observed compared to 100% mortality in the control insects (Fig 1C). Similarly, exposure to 0.6 ppm cypermethrin and 0.4 ppm deltamethrin for 3 days killed 55% and 45% of *CYP321A8* expressing insects respectively compared to 100% and 84% mortality in the control (Fig 1D and 1E). These data demonstrate that *CYP321A8* overexpression is sufficient to confer resistance to all three insecticides tested. To further prove that *CYP321A8* can metabolize these insecticides, *CYP321A8* and *Helicoverpa armigera* cytochrome P450 reductase (*CPR*) were co-expressed in Sf9 cells. Western blot and reduced CO-difference spectrum tests confirmed that *CYP321A8* was successfully expressed as a functional CYP450 enzyme (S2A and S2B Fig). Furthermore, assessment of the catalytic activity of recombinant CYP321A8 using the model substrate ECOD showed a specific activity of 0.68 pmol/min/pmol protein, confirming that the recombinant CYP321A8 is catalytically active (Fig 1F). The capability of recombinant CYP321A8 for metabolizing insecticides was evaluated by high-performance liquid chromatography (HPLC). After incubation for 1.5 h in the presence of NADPH, 51.9 ± 3.8% and 72.1 ± 9.0% of chlorpyrifos (Fig 1G), 18.8 ± 2.4% and 33.4 ± 3.0% of cypermethrin (Fig 1H), and 24.2 ± 2.8% and 37.7 ± 4.1% of deltamethrin (Fig 1I) were metabolized by 100 and 200 μg CYP321A8 respectively. No reduction in these insecticides was detected in the recombinant CYP321A8 that was incubated without NADPH. Proteins collected from the control Sf9 cells exhibited only trace levels of metabolism of these insecticides. Taken together these *in vivo* and *in vitro* functional analyses demonstrate that *CYP321A8* can confer resistance to chlorpyrifos, cypermethrin, and deltamethrin.

### Constitutive overexpression of *CncC*/*Maf contributes to* the overexpression of *CYP321A8* in the resistant strain

**Fig 2:**
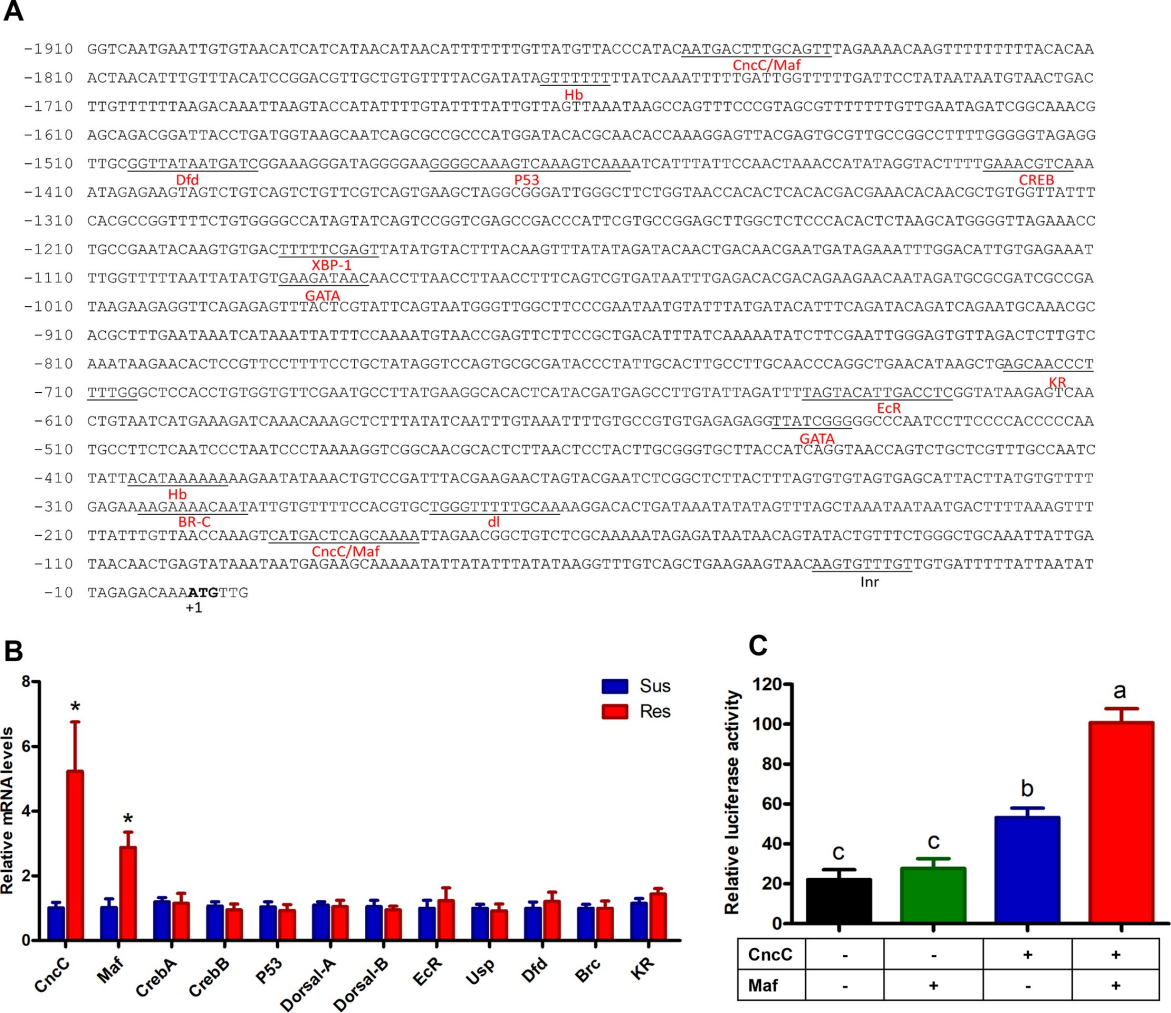
Overexpression of *CncC* and *Maf* increases the promoter activity of *CYP321A8*. (A) Prediction of transcription factor binding sites in a ~2 kb region of the promoter of *CYP321A8* gene. Nucleotides are numbered relative to the translation start site (ATG) indicated by +1. The predicted binding sites for transcription factors sites are underlined. The predicted transcription initiation site (Inr) is underlined. (B) Relative expression of transcription factors in the susceptible and resistant strains of *S*. *exigua* as determined by RT-qPCR. Error bars display SD. A significant difference (p < 0.05) in expression between the Sus and Res strains is indicated using an asterisk (ANOVA with post-hoc Tukey’s HSD). (C) The Luciferase activity in cells transfected with the reporter construct (the luciferase gene is under the control of CYP321A8 promoter) and CncC or Maf and both CncC and Maf expression constructs. Different letters above the bars indicate significant differences based on ANOVA followed by post-hoc Tukey’s HSD (p < 0.05).

As a first step towards the identification of the molecular mechanisms underpinning the overexpression of *CYP321A8* in the resistant strain, *in silico* analysis of the putative promoter sequence of this gene was conducted. Predictive analytics of TF binding sites (Fig 2A) showed binding sites for *CncC/Maf*, *Hb* (*Hunchback*), *Dfd* (*Deformed*), *P53*, *CREB (cyclin AMP response-element binding protein)*, *EcR (Ecdysone receptor)*, *BR-C (Broad complex)*, *XBP-1* (X-box binding protein 1), GATA binding, *Dl* (*Dorsal*) and *Kr (Krüppel)*. Analysis of expression levels of the TFs that bind to these predicted sites by RT-qPCR revealed that *CncC* and *Maf* genes were significantly overexpressed (5.2- and 2.8-fold, respectively) in the resistant strain (Fig 2B). However, no significant differences in expression of *CREA*, *CREB*, *Dfd*, *P53*, *Dorsal-A*, *USP*, *Dorsal-B*, *EcR*, *BR-C*, and *Kr* were observed between the two strains. To examine if *CncC*/*Maf* enhances the expression of *CYP321A8* in the resistant strain, open reading frames of these two genes were ligated into the expression plasmid and co-transfected with a 1900 bp fragment of the *CYP321A8* putative promoter ligated into the reporter gene vector. Maf slightly increased the promoter activity of *CYP321A8* (1.41 –fold, p-value = 0.052) and CncC significantly improved the promoter activity. A further increase was detected in the presence of both CncC and Maf (Fig 2C). These data thus provide evidence that the constitutive overexpression of *CncC* and *Maf* lead to an increase in the mRNA levels of *CYP321A8*.

**Fig 3:**
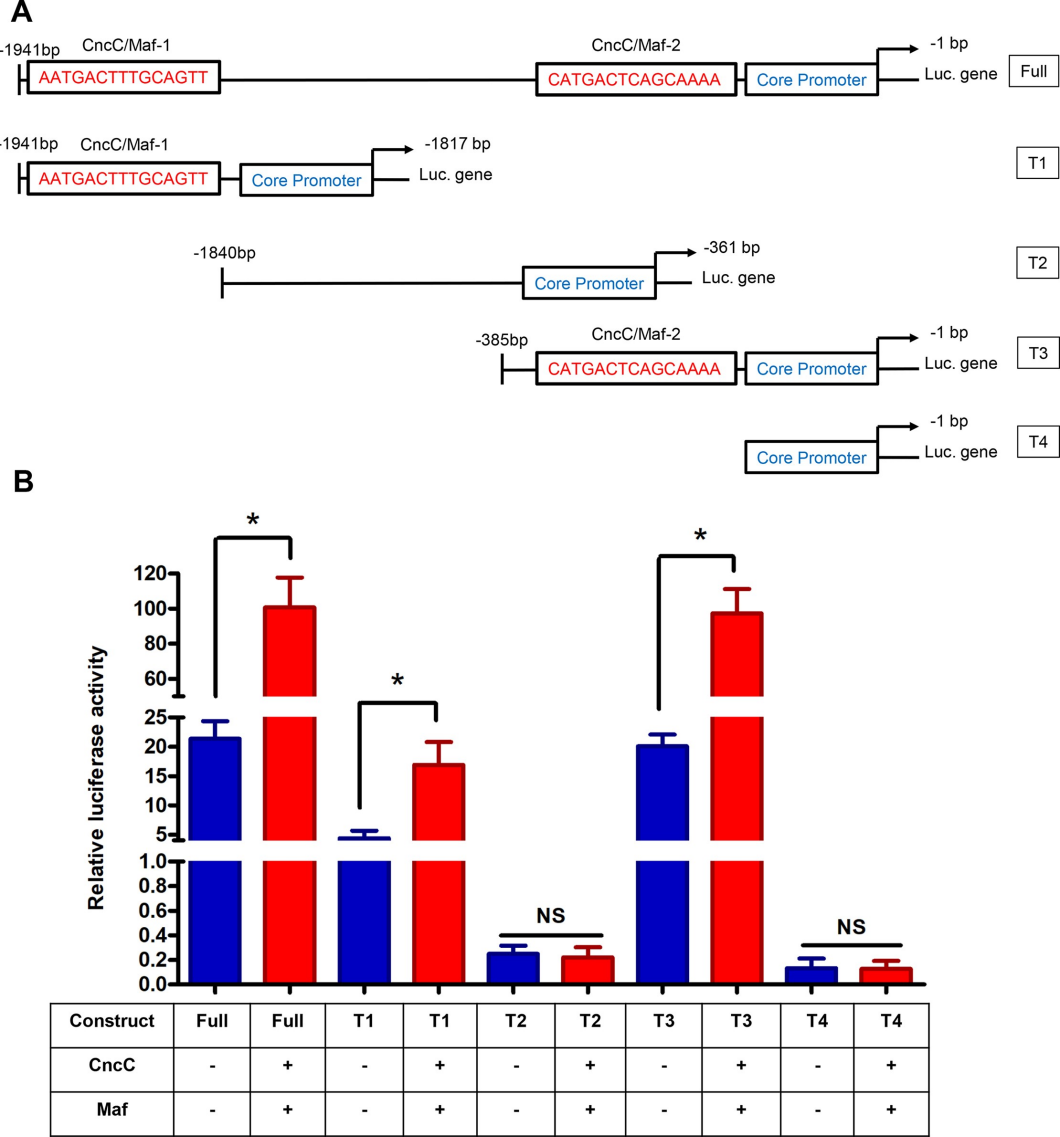
*CncC and Maf* regulate the expression of *CYP321A8* by binding to proximal and/or distal sites. (A) Schematic representation of the *CYP321A8* promoter deletions. (B) Analysis of the activity of *CYP321A8* promoter deletions in reporter gene assays in the presence and absence of CncC/Maf. The luciferase activity was normalized with the Renilla luciferase activity. Error bars display SD. The full-length promoter and each truncated version were compared using Student’s t-test. Significant differences (p < 0.05) in the luciferase activity are indicated using an asterisk.

**Fig 4:**
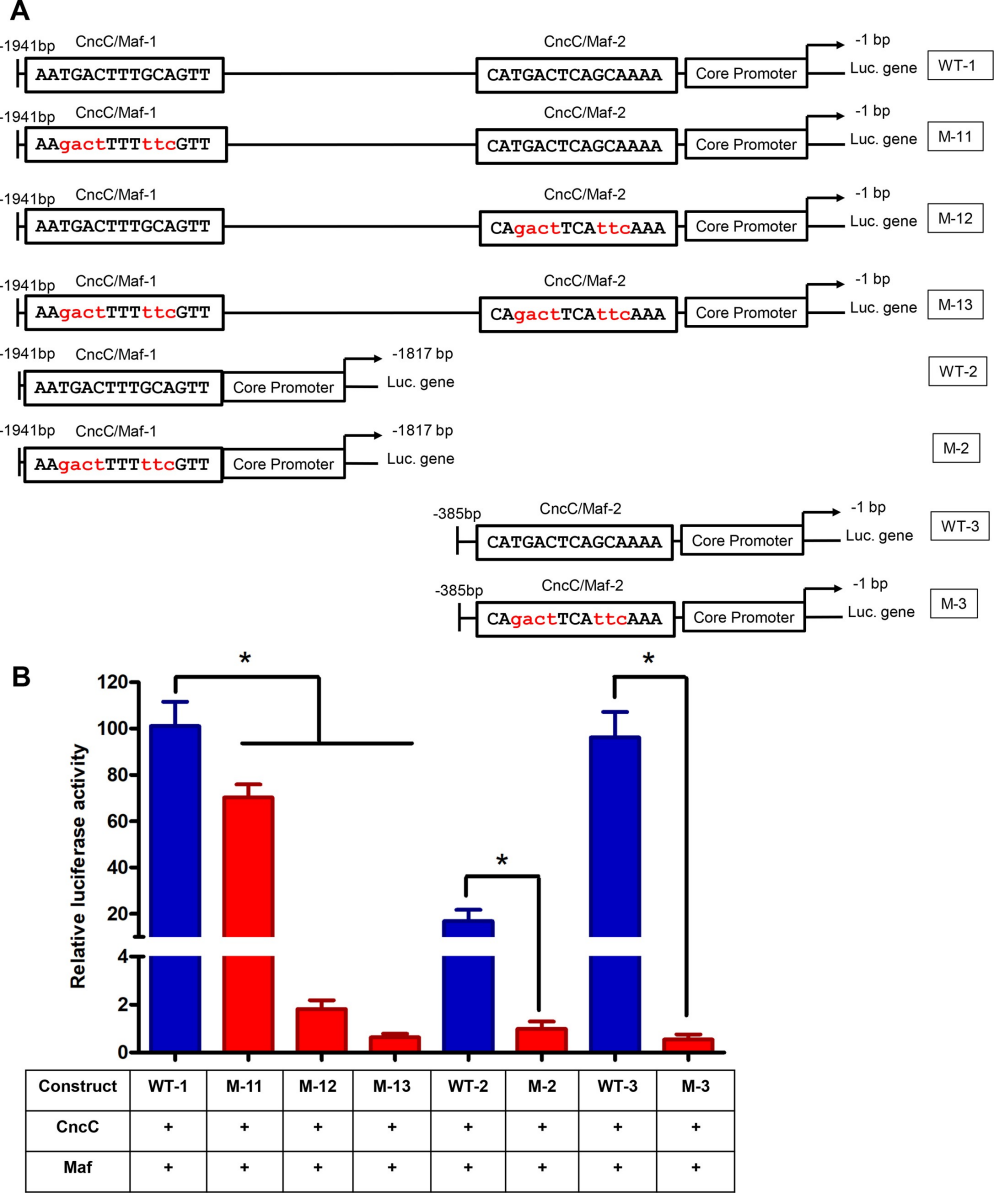
Mutations in *CncC/Maf* binding sites abolishes the ability of these transcription factors to induce the expression of *CYP321A8*. (A) Schematic representation of mutated *CYP321A8* promoter constructs. (B) The activity of these constructs in reporter assays. Error bars indicate SD. The activity of each mutated construct was compared with the corresponding wild-type control by Student’s t-test and significant differences (p < 0.05) are indicated with an asterisk.

To further determine the *CncC/Maf* binding sites in the *CYP321A8* promoter region, the promoter truncation constructs were prepared (Fig 3A). The full-length truncation contains the two *CncC/Maf* binding sites. T1 and T3 contain only the first and the second *CncC/Maf* binding sites, respectively. No *CncC/Maf* binding sites was presented in the T2 trunction and the truncation T4 contains only the core promoter. The activity of the full-length promoter (Full), -1941 to -1817 bp (T1) and -385 to -1 bp (T3) truncations was enhanced 4.71-, 3.85- and 5.97-fold respectively by CncC/Maf (Fig 3B). In contrast, CncC/Maf had no significant effect on the activity of -1840 to -361 bp promoter truncation (T2) or the truncation containing only the core promoter (T4). These data suggest that the promoter truncations, T1 and T3 contain the CncC/Maf binding sites. These results corroborated in silico analysis which predicted two putative CncC/Maf binding sites (AATGACTTTGCAGTT and CATGACTCAGCAAAA) located in the T1 and T3 truncation respectively. To confirm these sequence regions contain *bona fide* CncC/Maf sites we introduced mutations into the *CYP321A8* promoter at these positions using site-directed mutagenesis (Fig 4A). In the construct M-11, the first CncC and Maf binding site AATGACTTTGCAGTT was replaced with AAgactTTTttcGTT and the second site was not modified. In M-12, the second CncC and Maf binding site CATGACTCAGCAAAA was replaced with CAgactTCAttcAAA, and the first site was intact. Meanwhile, both of the two sites were mutated in the M-13. Only the first site AATGACTTTGCAGTT presents in the WT-2 and this was replaced with AAgactTTTttcGTT in the construct M-2. Mutated construct M-3 (CAgactTCAttcAAA) was obtained from the WT-3 containing only the second site (CATGACTCAGCAAAA). Compared to the wild type WT-1 construct, the promoter activity of the mutated constructs M-11, M-12, and M-13 was significantly reduced (Fig 4B). Similarly, the activity of the M-2 was significantly lower than the wild-type control WT-2. Compared to the WT-3 construct, the promoter activity of the mutated M-3 was reduced. These results thus confirm that the both of the two CncC and Maf binding sites play a significant role in the activation of *CYP321A8* promoter.

### A *cis*-acting mutation in the promoter of *CYP321A8* facilitates binding of the nuclear receptor, Knirps and contributes to upregulation of this P450 gene in the resistant strain

**Fig 5:**
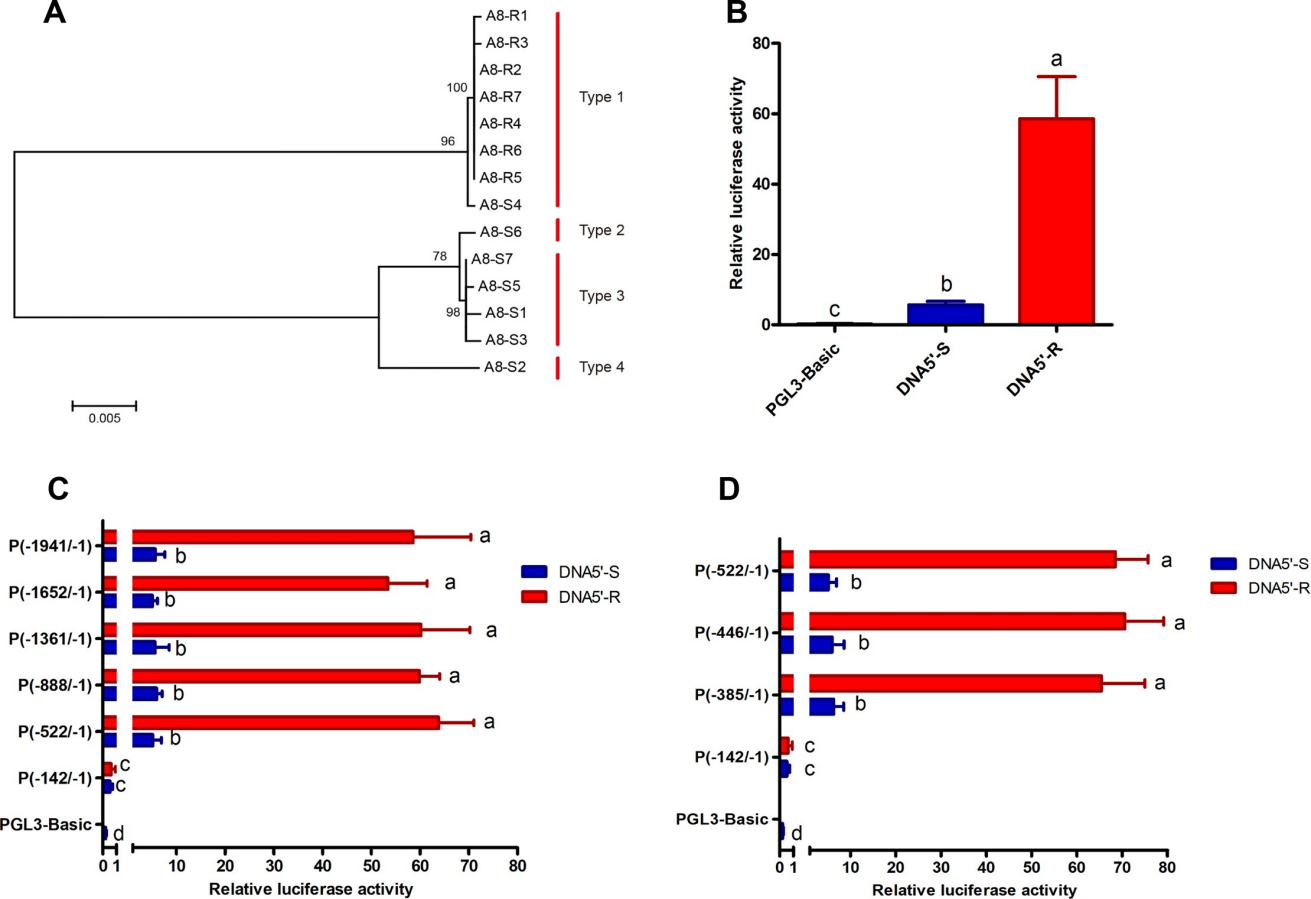
A *cis*-acting mutation in the promoter of *CYP321A8* enhances the expression of this P450 gene in the resistant strain of *S*. *exigua*. (A) Phylogenetic relationship of *CYP321A8* promoter sequences obtained from the susceptible and resistant strains. The phylogeny was inferred by the maximum likelihood and percentage bootstrap values from 1000 replicates are displayed. (B) Analysis of the activity of the *CYP321A8* resistant promoter (DNA5’-R) and susceptible promoter (DNA5’-S). Error bars display SD. Letters above bars denote a significant difference at p < 0.05 (ANOVA with post-hoc Tukey HSD). (C) The activity of progressive 5’ deletion constructs of the *CYP321A8* promoter. Error bars display SD. Letters to the right of bars denote significant difference at p < 0.05 (ANOVA with post-hoc Tukey HSD). (D) The activity of progressive 5’-deletion constructs from -522 to -142 of the *CYP321A8* promoter from the susceptible and resistant strains. Error bars display SD. Letters to the right of bars denote significant difference at p < 0.05 (ANOVA with post-hoc Tukey HSD).

**Fig 6:**
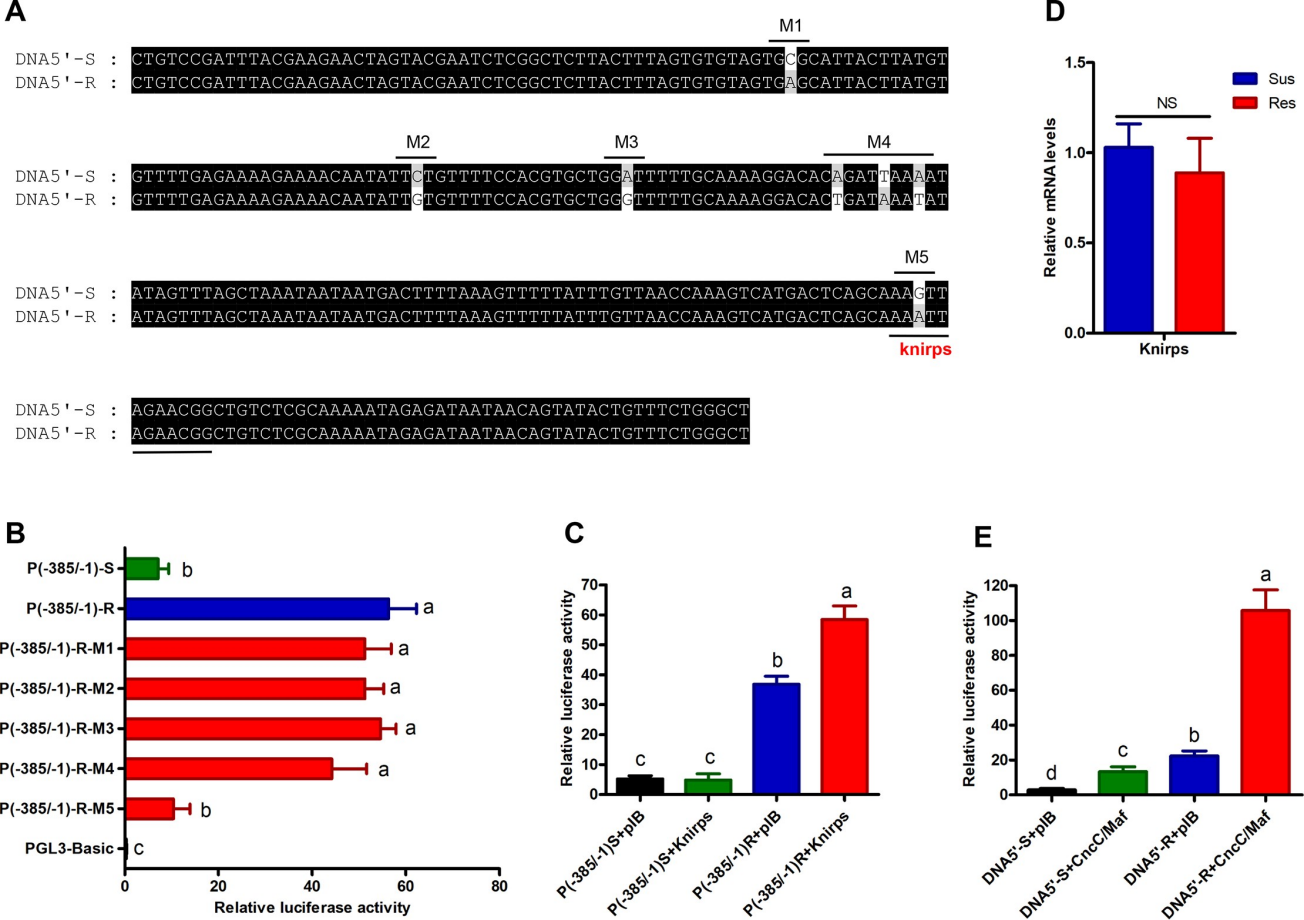
A *cis*-acting mutation in the *CYP321A8* promoter of the resistant strain facilitate binding of nuclear receptor, Knirps and contributes to upregulation of *CYP321A8*. (A) Alignment of a region of the *CYP321A8* promoter of the resistant and susceptible strains. Mutations at five positions are indicated above the alignment. (B) Activity of mutant constructs of the *CYP321A8* promoter from the resistant strain, where mutations in the region from -385 to -142 were reverted to wild-type. Error bars display SD. Letters to the right of bars denote significant difference at p < 0.05 (ANOVA with post-hoc Tukey HSD). (C) Activity of the promoter constructs P(-385/-1)S (susceptible strain) and P(-385/-1)R (resistant strain) in reporter gene assays in the presence and absence of Knirps. PIB represents the empty vector pIB/V5-His. Error bars display SD. Letters above bars denote significant difference at p < 0.05 (ANOVA with post-hoc Tukey HSD). (D) Relative expression of *Knirps* in the susceptible and resistant strains of *S*. *exigua* as determined by RT-qPCR. Error bars display SD. (E) The activity of the promoter constructs (DNA5’-R and DNA5’-S) in the presence and absence of *CncC/Maf* constructs. Error bars display SD. Letters above bars denote a significant difference at p < 0.05 (ANOVA with post-hoc Tukey HSD).

To examine the extent of genetic variation in the 5′-flanking region upstream of *CYP321A8* in the resistant and susceptible strains, a 1900 bp region of the putative promoter derived from seven individuals of each strain was cloned and sequenced. Phylogenetic analysis of sequence data showed that all of the putative promoter sequences from the resistant strain grouped into a single clade (Type 1, Fig 5A), suggesting a single *CYP321A8* promoter haplotype is found in this strain. In contrast promoter sequences from individuals of the susceptible strain formed four groups revealing a higher degree of genetic diversity in the *CYP321A8* putative promoter of this strain (Types 1–4, Fig 5A). A comparison of *CYP321A8* putative promoter sequences from the resistant and susceptible strains identified several mutations that differentiate the strains (S3 Fig). To determine if one or more of these mutations contribute to the observed increased activity of the putative promoter of the resistant strain, the putative promoter of this strain and the consensus promoter sequence of the susceptible strain were ligated into the vectors and assayed in Sf9 cells. As shown in Fig 5B, the *CYP321A8* promoter from the resistant strain showed a 10.4-fold (p-value < 0.05) higher activity compared with that in the susceptible strain. To identify the specific region of the promoter that is responsible for this enhanced activity, a series of truncations (-1941/-1, -1652/-1, -1361/-1, -888/-1, -522/-1 and -142/-1) were prepared. When these were tested in reporter gene assays, five (-1941/-1, -1652/-1, -1361/-1, -888/-1 and -522/-1) out of the six truncations tested showed significantly higher activity (10.4-fold, 10.6-fold, 10.7-fold, 10.1-fold and 12.1-fold, p-value < 0.05) than the promoter of the susceptible strain (Fig 5C). In contrast, the sixth truncation (-142/-1) showed no significant difference in activity between the resistant and susceptible strains (Fig 5C). These results suggest that the region responsible for the enhanced activity of the *CYP321A8* promoter of the resistant strain is in the 380-nucleotide region located between -522 and -142. To further characterize this region, two additional truncations (-385/-1 and -446/-1) were prepared and tested in Sf9 cells. As shown in Fig 5D, both the truncations maintained the difference in activity seen between promoters derived from the resistant and susceptible strains. These data suggest that the region responsible for the difference in the activity of the *CYP321A8* promoter in the resistant and susceptible strains is located in the 243-nucleotide region located between -385 and -142. The promoters of the resistant and susceptible strains are distinguished by five mutations in this region (Fig 6A). To identify which of these mutations are important for enhanced activity of the *CYP321A8* promoter in the resistant strain, the five mutations in the resistant promoter sequence were individually reverted to that in the susceptible promoter using site-directed mutagenesis. In reporter assays, one of the five mutations (M5, G > A at position -197bp) tested significantly reduced *CYP321A8* promoter activity (p-value < 0.05) (Fig 6B) suggesting that this mutation could be responsible for the elevated activity of the *CYP321A8* promoter in the resistant strain. Notably, the M5 mutation is located in a predicted binding site of the nuclear receptor, *Knirps*, suggesting this nuclear receptor may contribute to the increase in expression of *CYP321A8* in the resistant strain. To test this hypothesis, *Knirps* expression construct was prepared and transfected along with the *CYP321A8* promoter constructs into Sf9 cells. Expression of *Knirps* significantly increased the activity of the -385/-1 promoter construct from the resistant strain but not the corresponding construct from the susceptible strain demonstrating that the M5 mutation facilitates binding of Knirps leading to an increase in the activity of this promoter (Fig 6C). To examine if *Knirps*, like *CncC/Maf*, is overexpressed in the resistant strain, RT-qPCR was used to compare its mRNA level in the resistant and susceptible strains. No significant difference in the expression of this gene was observed between the two strains (Fig 6D). Thus, the *cis*-acting mutation that facilitates the binding of Knirps to *CYP321A8* promoter, but not the overexpression of *knirps* is responsible for the overexpression of *CYP321A8* in the resistant strain.

### *Trans* and *cis*-acting factors synergistically upregulate the expression of *CYP321A8 in the resistant strain*

To determine whether the overexpression of the *trans*-acting factors *CncC/Maf* and the *cis-*acting mutation in the promoter coordinately enhance the expression of *CYP321A8*, the constructs expressing *CncC/Maf* and *knirps*, and the *CYP321A8* promoter constructs with or without the Knirps binding site were co-transfected into Sf9 cells. Expression of *CncC/Maf* significantly increased the activity of both the *CYP321A8* promoter constructs with and without the Knirps binding site (p-value < 0.05) (Fig 6E). However, a much higher level of reporter activity (41-fold, p-value < 0.05) was observed in cells transfected with *CncC/Maf* expression constructs along with the construct containing the Knirps binding site when compared to the activity of the promoter construct without the Knirps binding site. Indeed, the increase in the reporter activity observed (41-fold greater than the wild-type promoter in the absence of CncC/Maf) was much greater than the sum of the individual effects of the *cis*-acting mutation and *CncC/Maf* overexpression (9.1-fold + 4.7-fold) revealing a synergistic interaction between these two mechanisms. These data clearly demonstrate that these *trans*- and *cis*-acting elements act synergistically to upregulate *CYP321A8* in the resistant strain.

## Discussion

Numerous studies have demonstrated the importance of cytochrome P450s in mediating resistance to insecticides in many insect and mite species [10]. Most of these studies have focused on the role of P450s in conferring resistance to a single insecticide, or a few insecticides belonging to the same class. Here we show that *CYP321A8* can contribute to resistance to multiple insecticides belonging to different chemical classes. The fact that *CYP321A8* metabolizes two pyrethroid insecticides (deltamethrin and cypermethrin) and the organophosphate chlorpyrifos, which have very different chemical structures, suggests that this P450 may be an important generalist enzyme that protects *S*. *exigua* from a range of xenobiotics. The related metabolites will be further identified. Just a handful of previous studies have unequivocally identified P450s capable of metabolizing structurally different insecticides belonging to different mode of action classes. In *D*. *melanogaster*, overexpression of *CYP6G1* was shown to confer resistance to four unrelated insecticides (diazinon, nitenpyram, lufenuron and DDT)[1,29]. Similarly, *CYP6CM1* of *Bemisia tabaci* can detoxify several neonicotinoid insecticides and the feeding blocker pymetrozine[30,31]. Finally, *CYP6M2*, overexpressed in resistant populations of *A*. *gambiae*, can metabolize pyrethroids, carbamate and DDT from three different chemical classes[32]. Together with the results of our study, these findings illustrate the remarkable flexibility of insect P450s in metabolizing chemically diverse insecticide substrates.

While the capacity of P450s to metabolize insecticides to protect insects from insecticides is well established, precisely how the P450s that are overexpressed in resistant insects are regulated is considerably less well understood. In the current study, we showed that the transcription factors *CncC*/*Maf* are overexpressed and bind to two xenobiotic response elements (XREs) from the *CYP321A8* promoter in the resistant strain. Several previous studies on this topic have also reported constitutive overexpression of these transcription factors in insecticide-resistant arthropod strains. For example, *CncC*/*Maf* were found to be overexpressed in a *Tetranychus cinnabarinus* strain with resistance to fenpropathrin, and controlled the enhanced expression of *CYP392A28*, *CYP391B1* and *CYP391A1* and resulted in resistance [33]. Similarly, *CncC* was shown to be upregulated in a deltamethrin-resistant *T*. *castaneum* strain and serve as an important regulator of multiple P450s belonging to the CYP6BQ subfamily that confer resistance to deltamethrin [34]. *CncC* and *Maf* were also found to be associated with *cis*-regulation of *CYP6P9a* and *CYP6P9b* in the malaria vector *Anopheles funestus* [17,18]. Finally, *CncC* and *Maf* regulate the overexpression of four P450 genes involved in imidacloprid resistance in *L*. *decemlineata* [35]. These studies, together with our findings, clearly demonstrate an important role for *CncC/Maf* as *trans*-regulators of P450 genes in arthropods. Furthermore, as suggested by the previous studies, their constitutive overexpression in resistant arthropods may lead to changes in the expression of multiple P450s. Our results are consistent with this, in which 30% of the P450s tested upregulated in the resistant strain. Together these findings suggest that constitutive overexpression of *CncC/Maf* may provide a mechanism for arthropod pests to more rapidly evolve insecticide resistance. Specifically, by increasing the expression of multiple P450s, the chances of upregulating a P450 that has the capacity to metabolize insecticide are significantly improved. Conversely this strategy may have a greater fitness cost than a *cis*-acting mechanism due to its effect on multiple genes, the overexpression of which would incur a metabolic cost. The mutation(s) leading to the constitutive overexpression of *CncC* and *Maf* in *S*. *exigua* and the arthropod species detailed above have not been identified. Given the conservation of this mechanism in a range of resistant arthropods, characterization of the genetic alterations leading to the upregulation of these transcription factors should be a priority of future studies. In this study, the *CncC/Maf* binding sites were identified. In combination with the previous studies [34], these data bring us closer to understanding the consensus motifs to which these important transcription factors bind, and the extent of their variability across insects. Such knowledge is important as it improves the accuracy of in silico prediction of the binding sites of these transcription factors as well as the P450s that may be regulated by them.

In addition to characterizing the role of these important *trans*-acting factors in regulating *CYP321A8* we also identified a *cis*-acting mutation in resistant *S*. *exigua* that contributed to an increase in the expression of this P450 gene. Although insertions/deletions or mutations in the promoter region are often implicated in resistance phenotypes that overexpress a gene encoding detoxifying enzyme, only a few studies have analyzed this in detail. For example, a single nucleotide substitution located near the transcription start site of *CYP9M10*, increased the transcription of this gene in *Culex quinquefasciatus*[36]. In *Myzus persicae*, the expansion of a dinucleotide microsatellite in the promoter region resulted in the overexpression of *CYP6CY3* conferring resistance to neonicotinoid insecticides and nicotine[37]. Multiple mutations in the promoter of *CYP6FU1* enhanced its expression leading to deltamethrin resistance in *Laodelphax striatellus*[38]. Finally, the changes of *cis*-regulatory elements drive the overexpression of the CYP6P9a and CYP6P9b associated with the pyrethroid resistance in the major African malaria vector *An*. *funestus*[17,18]. Despite the important insights provided by these studies, the specific transcriptional activator(s) that act in concert with the *cis*-acting mutations were not identified. In contrast, in this paper, we demonstrate that the mutation in the *CYP321A8* promoter of resistant *S*. *exigua* upregulates this P450 gene by creating a binding site for the nuclear receptor knirps. These results advance fundamental knowledge of how the mutation in the promoter of P450 gene allow binding of a nuclear receptor that contributes to overexpression of this gene. In addition, identification of a mutation in the resistant strain compared with the susceptible strain of *S*. *exigua* also has applied importance. Specifically, it can be used as a marker to determine the frequency and distribution of resistance conferred by this mechanism and thus inform resistance management strategies [17,18]. In insects, the *knirps* gene encodes an orphan nuclear hormone receptor, which plays a vital role during its growth and development [39–42]. Here, we found that it might regulate the expression of P450 gene in *S*. *exigua*. Phylogenetic analysis of sequence data showed that the susceptible sequence A8-S4 gathered into the clade of resistant sequences, which might result from genetic diversity, but there is no knirps binding site in A8-S4.

**Fig 7:**
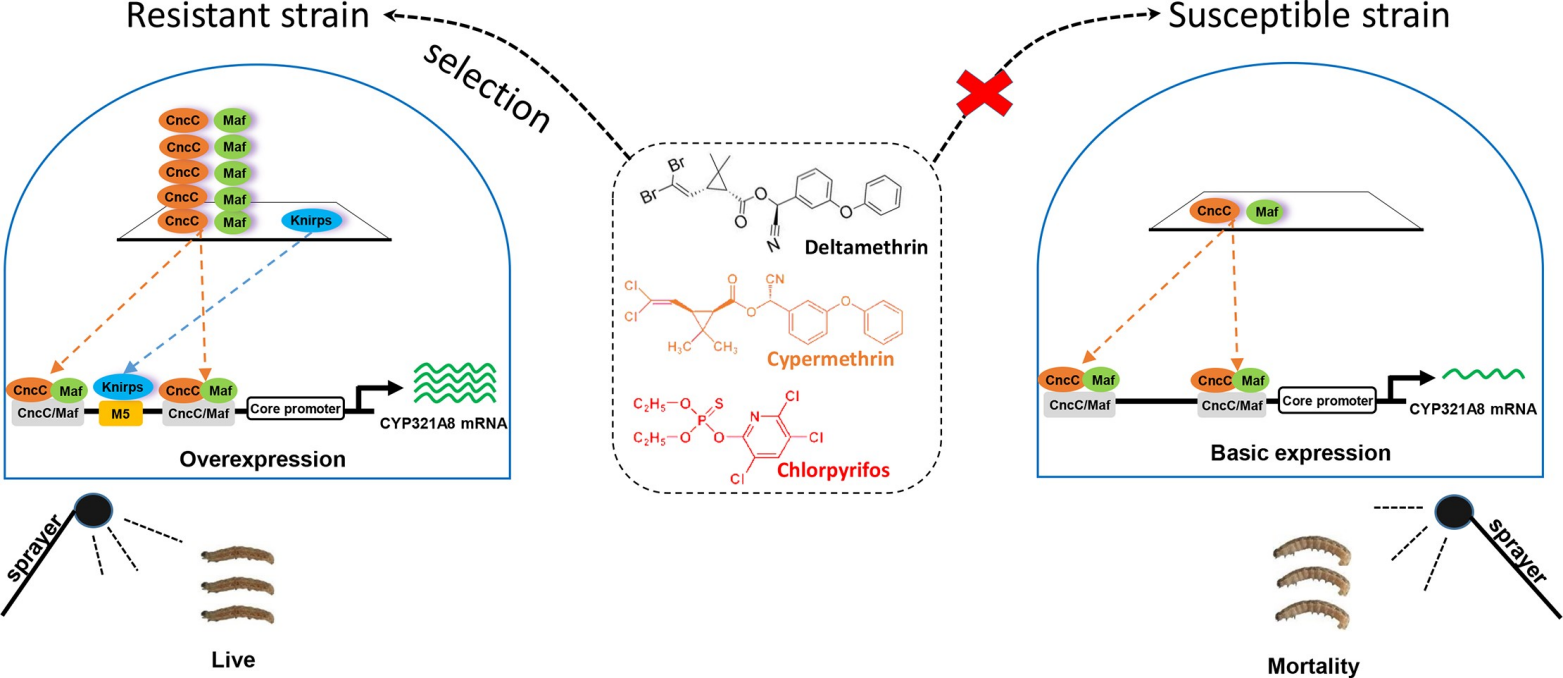
Schematic of the regulation of *CYP321A8* by *cis*- and *tran*s-acting factors. In the resistant strain, constitutive overexpression of the *trans*-acting factors *CncC* and *Maf* act in combination with a *cis*-acting mutation in the promoter of *CYP321A8*, which facilitates binding of nuclear receptor, Knirps, enhancing the expression of *CYP321A8* leading to insecticide resistance. In the susceptible strain, *CncC* and *Maf* are expressed at lower levels and there is no binding site for Knirps therefore, *CYP321A8* expression is low making them susceptible to insecticides.

The relative importance of *cis*- and/or *trans*-acting factors in regulating P450s associated with resistance, and how these mechanisms interact, is unclear. In this study, we demonstrate that the constitutive upregulation of *CncC* and *Maf* acts in combination with the *cis*-acting mutation in the promoter of *CYP321A8* to cause its overexpression in the resistant strain (Fig 7). In this regard, our findings parallel the results of the previous reports that suggested both *cis*- and *trans*-acting factors can act together to increase the expression of P450s that contribute to resistance. A 15 bp insertion, which disrupts a transcriptional repressor *mdGfi-1* binding site in the *CYP6D1* promoter, increased the expression of the *CYP6D1* gene by approximately 10-fold in the pyrethroid-resistant housefly strain [43]. Further studies revealed that the expression of *CYP6D1* is also regulated by a *trans*-acting factor coded by a gene located in chromosome 2, however, this factor has not been identified[44]. Similarly, an intact *Nrf2/Maf* binding site and an unknown trans-acting factor on chromosome 3 result in constitutive overexpression of *CYP6A2* involved in DDT resistance in *D*. *melanogaster* [13,26,45]. While these studies suggest that regulation of resistance genes by a combination of both *cis*- and *trans*-acting factors may be more common than previously appreciated, exactly how these factors interact to modulate P450 gene expression has remained unclear. Our data provide new information on this question by revealing that the *cis*- and *trans*-acting factors have a synergistic effect on *CYP321A8* expression in a chlorpyrifos-resistant strain of *S*. *exigua*. These TF binding sites will be further identified in field-collected samples. As the combined effect of these regulatory factors is much greater than the sum of their individual effects the evolution of both mechanisms would provide the better fitness benefit to *S*. *exigua* in the presence of insecticide.

In conclusion, we identified two independent mechanisms (constitutive overexpression of b-Zip transcription factors and mutation in the P450 promoter creating a *cis*-regulatory element that facilitates binding of a nuclear receptor) that work in concert to increase expression of a P450 gene and enhance resistance to insecticides in a major pest. The information on mechanisms of metabolic resistance could help to understand the development of resistance to insecticides by other pests and contribute to programs aimed at managing insecticide resistance.

## Materials and methods

### Insect strains

The chlorpyrifos resistant strain of *S*. *exigua* was collected from Welsh Onion, *Allium fistulosum*, in Huizhou, Guangdong province, China and the susceptible strain of *S*. *exigua* was obtained from Wuhan Kernel Bio-pesticide Company, Hubei, China. Larvae were reared on an artificial diet at 25°C under a 16-h light/8-h dark photoperiod with a relative humidity of 60 ± 5% [46].

### Insecticides

Cypermethrin (96.5% TG), deltamethrin (98% TG) and chlorpyrifos (96.5% TG) were purchased from Jiangsu Yangnong, Nanjing Red Sun and Nanjing Keweibang Co., Ltd, respectively.

### Toxicology bioassays and synergism assays

The leaf dip method was used to assay the toxicity of insecticides to *S*. *exigua* as described previously [46]. Each treatment needed at least 30, 3^rd^-instar larvae and the assays were repeated three times. Preliminary experiments were conducted to identify the dose of synergists that showed no detrimental effects on in 3rd instar larvae. 100 mg/L of PBO had no effects on larval survival and therefore, this concentration was used in synergism assays. Synergism ratio was obtained by analyzing differences between LC_50_ of insecticide alone and LC_50_ of insecticide with the synergist.

### Extraction of DNA and RNA

RNA was isolated using TRIzol Reagent (TaKaRa, Japan). The quantity of RNA was determined by a NanoDrop 1000 Spectrophotometer. The cDNA was synthesized by the HiScript 1st Strand cDNA Synthesis Kit (Vazyme, China) following the manufacturer’s instructions. DNA was obtained using an Insect DNA Kit (Omega, USA).

### Quantitive Real-Time PCR (qRT-PCR)

In order to analyze the relative expression level of CYP450 genes between the resistant strain and susceptible strain, the qRT-PCR was done with SYBR Premix Ex Taq (TaKaRa, Japan) [47]. 68 P450s were chosen according to our previous study [47]. The primers were designed using Primer 5 software and shown in S2 and S3 Tables. Primer specificity and PCR efficiencies were assessed by melting curve analysis and standard curves, respectively. The relative mRNA levels were calculated by reference genes *GAPDH* and *β-Actin*, and 2^−ΔΔCT^ method [48,49].

### Cloning of transcription factors

To identify the molecular mechanisms underpinning the overexpression of CYP321A8 in the resistant strain, sequences of six transcription factors and nuclear receptors (*CrebA*, *CrebB*, *P53*, *Dorsal-A*, *Dorsal-B*, and *Kr*) were chosen based on the predicted transcription factor binding sites and obtained from the transcriptomes of *S*. *exigua* [50]. The full-length sequence of these genes (MK302135-MK302140) was obtained by 5’-RACE and 3’-RACE using SMART RACE cDNA Amplification Kit (Clontech, USA). All PCR products were incorporated into the PMD-19T vector (TaKaRa, Japan) and identified by sequencing. All primers are shown in S4 Table. Sequence information of five transcription factors (*CncC*, *Maf*, *USP*, *Dfd*, and *Brc*) was included in our recent publication[51] and the sequence of *ecdysone receptor* was downloaded from NCBI (GU296540).

### Bioassays of transgenic *D*. *melanogaster*

In addition to characterizing the role of CYP321A8 in insecticide resistance, the UAS-CYP321A8 strain was constructed according to the previous methods [52]. The transgene CYP450 was expressed by crossing with the Act5C-GAL4 strain. The qRT-PCR and RT-PCR were used to confirm the expressions of the CYP321A8 gene in transgenic *D*. *melanogaster* by using the gene-specific primers (S5 Table). For insecticide bioassays, the F_1_ males were used and the offspring from the Act5C-GAL4 and w^1118^ strain was used as a control. Ten male flies (2-5-day-old) were added to each vial with 10 ml corn meal medium containing 0.6 mg/L permethrin, 0.05 mg/L chlorpyrifos and 0.4 mg/L deltamethrin. At least 6 replicates were used for each experiment.

### Expression of CYP321A8 in Sf9 cells and functional assay

To determine whether CYP321A8 can metabolize insecticides in vitro, the full-length *S*. *exigua CYP321A8* and *H*. *armigera CPR* gene sequences were downloaded from NCBI (KX443441 and KF419215). The primers were designed to amplify the gene open reading frames [53] (S5 Table). These two genes were cloned into pFastBacHTA vector (Invitrogen, USA) and transferred into DH10 Bac cells. The Bacmid DNAs were purified and transfected into Sf9 cells by Cellfectin II Reagent (Thermo Fisher Scientific, USA). The virus titer was determined using a plaque assay [54]. For expression, Sf9 cells were co-infected with baculoviruses of the CPR and CYP450 with a multiplicity of infection of 2: 0.2. To compensate for low levels of endogenous hemin, the culture media were supplied with precursor hemins. Cells were cultured for 48 h, then harvested and washed two times, and the microsome was collected following the protocol and kept at -80°C [55]. The expressed P450 content was examined using CO-difference spectra [56] and western blot by 6×his tag antibody (Abcam, UK). Total protein concentration was determined according to Bradford technique [57], and the reduction of cytochrome c was used to estimate the activity of CPR [58].

P450 monooxygenase activity was estimated by measuring ethoxycoumarin-O-deethylase (ECOD) activity assay as described previously [59]. Sixteen 3rd-instar larvae were homogenized and microsomal fraction was obtained as described previously [46]. The O-dealkylation of recombinant P450s was identified by using ECOD activity assay. The assay was performed as described previously by Shi et al[53]. Microsomes and ECOD substrate were incubated for 5 min before adding CuOOH. The fluorescence was measured in the SpectraMax M5 multimode reader at 380 nm excitation, 460 nm emission and 30°C for 15 min. P450 activities were counted based on the standard curve of 7-hydroxycoumarin and expressed as mean pmoles of 7-OH per mg or pmole of microsomal protein/min ± SD.

HPLC analysis of insecticide metabolism

For chlorpyrifos, cypermethrin and deltamethrin metabolism studies, the in vitro reactions were performed according to our previous approach [52]. The non-insertion microsomes (empty plasmid) were used as the controls. The insecticides were extracted using 500 μL Acetonitrile, then centrifuged at 16000 g for 18 min. Finally, 200 μL of the supernatants were injected to the HPLC and checked immediately using C18 column (4.6×250 mm, 5 μm; Ameritech Technology, USA) with 82% acetonitrile, 90% methanol and 80% methanol as the mobile phase for deltamethrin, cypermethrin and chlorpyrifos, respectively, and a flow rate of 1 ml/min. The quantities of deltamethrin, chlorpyrifos, and cypermethrin which remain in the samples were detected at 240, 289 and 230 nm wavelength, respectively.

### Cloning of the CYP450 5’-flanking regions and Luciferase reporter assays

To obtain the promoter sequence of *CYP321A8*, four different restriction enzymes were used for digesting the Genomic DNA according to the Universal Genome Walker Kit (Clontech, USA). Then, the digested genomic DNAs were ligated to the adaptors, and the target sequences were amplified with the LATaq mix (Vazyme, China). All the primers are shown in the S6 Table. The fragments were ligated to the PMD19-T vectors and identified by sequencing. The ALLGEN and JASPAR software with the ‘Insecta’ group was used to obtain the putative TF sites [38,60]. Promoter region sequences (DNA5’-R and DNA5’-S) were amplified and ligated to the PGL3-Basic vector (Promega, USA) (Accession nos: MK327547 and MK327548). Various promoter truncations of *CYP321A8* were obtained using the full promoter plasmid as the template. Mutated promoter plasmids were obtained following [61]. The over-expression constructs of transcription factors were produced by cloning the ORF into the pIB/V5-His vector. All primers are displayed in S7 Table.

Sf9 cells were cultured in 24-well cell plates at 4 x 10^5^ cells density. The promoter constructs and expression plasmids were transfected according to our previous approach [52]. 1 μg promoter constructs (DNA5’-R, DNA5’-S, truncations and mutated truncations) and 0.02 μg pRL-CMV were co-transfected to the cells with the help of 2 μL of FUGENE transfection reagent. The medium for transfection was replaced with 500 μL complete medium after incubation for 4h. Finally, the cells were collected after 48 h and luciferase activities were measured [52].

### Statistical analysis

The data analyses were performed using SPSS 16.0 software (SPSS, USA). The differences between two samples were analyzed by Student’s t-test. While differences among more than two samples were analyzed using one-way ANOVA with Tukey’s HSD test. The significant difference was set at P-value < 0.05.

## Supporting information

S1 Table Resistance and synergism of PBO in insecticide resistant strain of *S. exigua*. (DOCX)

S2 Table Primers used in quantitative real-time PCR of Cytochrome P450 genes. (DOCX)

S3 Table Primers used in quantitative real-time PCR of transcription factors. (DOCX)

S4 Table Primers used for gene amplification of transcription factors. (DOCX)

S5 Table Primers used for Constuction of transgenic *Drosophila* and eukaryotic expression. (DOCX)

S6 Table Primers used for cloning 5’-flanking regions. (DOCX)

S7 Table Primers used for reporter and promoter constructs. (DOCX)

S1 Fig Relative expression of the CYP321A8 transgene in the transgenic *D. melanogaster* Act5C-CYP321A8 strain and the control sample with no transgene expression. PCR was performed using the synthesized cDNA as a template and primers specific to CYP321A8 (A). In addition, the relative expression levels of the CYP321A8 transgene were assessed by qRT-PCR in the F1 progeny under the Act5C driver (B). The data shown are the mean ± standard error of the mean (n = 3).
(TIF)

S2 Fig CYP321A8 expression and functional analysis. A) Western blot analysis of CYP321A8 expression in microsomes prepared from baculovirus-infected Sf9 insect cells. (B) Reduced CO-difference spectrum of the recombinant CYP321A8.
(TIF)

S3 Fig The alignment of upstream sequences of *CYP321A8* gene from susceptible and resistant strains of *S. exigua*. The nucleotides are numbered relative to the translation start site (ATG), with sequence upstream of it preceded by “-“.
(TIF)
