# Supplementary material for: Changes in both trans- and cis-regulatory elements mediate insecticide resistance in a lepidopteron pest, Spodoptera exigua
Source: PLoS Genet. 2021 Mar 9;17(3):e1009403. doi: 10.1371/journal.pgen.1009403 (PMC7978377; doi:10.1371/journal.pgen.1009403)
Supplement: S1 Table — (DOCX) [file pgen.1009403.s001.docx]

###### **Table S1 Resistance and synergism of PBO in insecticide resistant strain of *S. exigua***

| Strains | Insecticides | LC_50_:mg AI/L (95%FL) | Slope ± SE | | χ^2^ (df) | | RR^a^ | | | SR^b^ | | |  |
| --- | --- | --- | --- | --- | --- | --- | --- | --- | --- | --- | --- | --- | --- |
| Sus | Chlorpyrifos | 2.019 (0.856 - 6.105) | 0.914 ± 0.299 | | 0.834 (3) | |  | | |  | | |  |
|  | Chlorpyrifos + PBO | 1.901 (0.966 - 3.569) | 1.285 ± 0.347 | | 1.444 (3) | |  | | | 1.1 | | |  |
| Res | Chlorpyrifos | 1888.563 (993.749 - 4277.735) | 1.390 ± 0.455 | | 0.374 (3) | | 935 | | |  | | |  |
|  | Chlorpyrifos + PBO | 233.344 (93.484 - 400.376)* | 1.063 ± 0.280 | | 0.361 (3) | | 116 | | | 8.1 | | |  |
| Sus | Cypermethrin | 1.448 (0.683 - 2.786) | 1.111 ± 0.303 | | 0.153 (3) | |  | | |  | | |  |
|  | Cypermethrin + PBO | 1.097 (0.469 - 1.962) | 1.197 ± 0.314 | | 0.379 (3) | |  | | | 1.3 | | |  |
| Res | Cypermethrin | 816.384 (485.142 - 1186.413) | 1.421 ± 0.223 | | 0.904 (3) | | 563 | | |  | | |  |
|  | Cypermethrin + PBO | 226.683 (102.266 - 358.142)* | 1.273 ± 0.247 | | 0.334 (3) | | 156 | | | 3.6 | | |  |
| Sus | Deltamethrin | 2.022 (1.096 - 5.836) | 0.194 ± 0.290 | 0.111 (3) | |  | | |  | | |  |  |
|  | Deltamethrin + PBO | 1.413 (0.921 - 2.154) | 1.462 ± 0.319 | 1.640 (3) | |  | | | | | 1.4 | | |
| Res | Deltamethrin | 615.981 (333.405 - 1704.856) | 0.722 ± 0.143 | 3.044 (3) | | | | 305 | | |  | | |
|  | Deltamethrin + PBO | 208.390 (122.283 - 270.129)* | 0.910 ± 0.219 | 1.934 (3) | | | | 104 | | | 3.0 | | |

^a^ RR, Resistance ratio

^b^ SR, Synergistic ratio

* Significant difference with insecticide only without synergist (PBO)
