# Supplementary material for: Changes in both trans- and cis-regulatory elements mediate insecticide resistance in a lepidopteron pest, Spodoptera exigua
Source: PLoS Genet. 2021 Mar 9;17(3):e1009403. doi: 10.1371/journal.pgen.1009403 (PMC7978377; doi:10.1371/journal.pgen.1009403)
Supplement: S2 Table — (DOCX) [file pgen.1009403.s002.docx]

**Table S2 Primers used in quantitative real-time PCR of Cytochrome P450 genes**

| Name | Sense primer | Anti-sense primer | Amplicon  (bp) | qRT-PCR  efficiency (%) |
| --- | --- | --- | --- | --- |
| CYP4G75 | CCGACTTCAAACTGCAAGCT | AATCAACACGCCTTTGCCAA | 94 | 100.09 |
| CYP4G74 | CTGTCGACCATCCTCCGTAA | CTAAGCGACGTTGGGGATTG | 150 | 97.34 |
| CYP4L15 | GCATTGGTCAAAAGTTCGCG | TCTGGTTCCTGAGCTGATGG | 94 | 94.92 |
| CYP4L9 | GCATTTCGTACACGCTCTA | GTTCCTGGTAAGTTGGGTC | 117 | 98.12 |
| CYP4L7 | ACTGCAGACAATGTTCGGTG | CGCATCGACTCCTTAATGACC | 99 | 97.23 |
| CYP4M18 | GACCTGTATCCCAACCCGAA | AATTTCTTGGCCCTGCACTG | 109 | 94.87 |
| CYP4M17 | GTAACCACTCAATTCCCGCC | AACACTTCAGGGTTCGGGAA | 91 | 93.57 |
| CYP4M15 | GCAGACACGCAGGAAGATTC | TCTAGGAACCGTTCGCTGTT | 96 | 98.89 |
| CYP4M14 | CGGGCATGAAGTTTGGTGAA | AGTGGAAAGCTGGAGTGAGG | 129 | 97.90 |
| CYP4S8 | ACAACTCCTCTCGTGGGAAG | ATTCTGAGCTCTTTGGGCCT | 101 | 95.67 |
| CYP4S9 | TGGTGGTGTACTGATGGGTC | ACGTCATCAGCGAACGAATG | 126 | 94.35 |
| CYP6AB61 | CACTCAAGGGGCCTCACTTA | CACAAGTCACCGTCAGCAAA | 80 | 98.99 |
| CYP6AB12 | GGAAAGCCAGTATGACGCAG | AGAGCGAAGAAATCCGACGA | 80 | 101.1 |
| CYP6AB31 | AGCAGAAGGGAGTGATGGTC | CCAGCCGCGAAGAATACAAA | 127 | 99.98 |
| CYP6AB14 | TCTTGATGCTGACTCGCTCA | TACAGGCTTCCGGGAACATT | 125 | 95.46 |
| CYP6AE10 | GGACAATGGTGAAGACTGGC | TGCGACAAACTTGAGTGCTC | 91 | 97.24 |
| CYP6AE97 | GGATGATGAGCTGCTGGTTG | GCATGAACTCGTCGACTTCC | 149 | 98.45 |
| CYP6AE47 | GCATGGAGTCGATGCGTATT | TCCAAGGACCGGGTAGAAAC | 121 | 104.7 |
| CYP6AE74 | GTGGGAGACAGCATCAGGAA | ACCCAGCAGCAAAGAAAACA | 109 | 105.2 |
| CYP6AE68 | AGTATGTCGAATATGAAGGGTG | CGTAGTCGCCGATGTCTC | 120 | 94.89 |
| CYP6AE70 | ATCTCACGCCTCTGTTCTC | GTCAAAGTCCTCGTATCAA | 196 | 96.77 |
| CYP6AN4 | GTTCCCTCTCGTAGTGCAGT | GTGGTGAAGCGAGCCATTAG | 108 | 98.22 |
| CYP6B31 | AACGTCTGATGTGATTGCGG | CTTCTTCTGGACGTCTGGGT | 120 | 97.34 |
| CYP6B68 | AGAATACAAAGTGCCCGGGA | CAGGGTTGAATTGTTCGGGG | 119 | 98.47 |
| CYP6B50 | TGTGAGAGAACTGCATCCCT | GGAGCTGTGCGAACTTTGAA | 83 | 97.89 |
| CYP18A1 | CTTATCGCCAACCTTCACCG | GTTATGCCGCAGATGACGTT | 92 | 93.99 |
| CYP18B1 | ATTCAGCAACGCGAACAGTC | ACGGGATCAACATCTGGGAA | 140 | 92.98 |
| CYP301A1 | GCGGATGGTGCCTATAATCG | TCTTCCAATCAAGCCGCCTA | 112 | 96.84 |
| CYP301B1 | CCGACAAATAAGGGATGAA | GTCTGCACTGCGTGAATAA | 165 | 94.95 |
| CYP302A1 | CCTCTCGGATGCCACAGTAT | TATACATGACCGTGGCCCAA | 134 | 100.2 |
| CYP304F1 | ACTGACGGTTACTTCTGGCA | CATTTCAGCGGGGTACTTCG | 162 | 102.6 |
| CYP305B1 | TATCCCGTAGTCTGCATGGC | CCACAACTCTGGGTCGAAGT | 129 | 99.12 |
| CYP306A1 | AGATCCAAGAAGAGGGGTGC | TCTGAGCTTGACCACGTGTT | 126 | 98.72 |
| CYP307A2 | GGCTCGCACCATTCTACAAG | AGTCCTTTTCTGGTCCCTCG | 132 | 99.01 |
| CYP314A1 | CCTACGTTGGTACTGGTGGT | TACTCCAGAAAAGCCACCGT | 145 | 97.56 |
| CYP315A1 | AGCTGGTGATACGACGTCTT | GCGCAACGGGATATAACCTC | 143 | 99.21 |
| CYP321A16 | GCCGTCGGTATAGGTCAGTT | CAATGCTTTCCACTCCTCGG | 118 | 100.6 |
| CYP321A9 | CAGAAAGGAACCAGCCATCG | CCTTCGACGCTTTCCATACC | 135 | 103.4 |
| CYP321A8 | AAACAACCCCAAGACGCATG | CCAATGCCAAAGATAGCCCC | 90 | 105.6 |
| CYP321B1 | TGGAGCCTACAGACGAGTTG | TCCAAAAGTTCCGGCAGTTG | 86 | 91.99 |
| CYP321B4 | TTCACGACCTGGGACTGTAC | TGGACCGTTGAGCATTAGGA | 143 | 102.7 |
| CYP324A1 | GTCCTGGTCCAGCATCTTCT | CCTCTCTGTTCCACCACTGT | 120 | 95.67 |
| CYP332A1 | CTGAAAGATCTTGCCTCGCC | CCTCTCTGTTCCACCACTGT | 110 | 94.89 |
| CYP333A12 | TGCGAGCGTGTTTGAAAGAA | TGGGGCTATTACATCCACCC | 125 | 93.27 |
| CYP333B4 | CACGTTGCTCACACTACTGG | TGTCCTGAGCCACTTCTTCA | 139 | 90.88 |
| CYP333B40 | GCAGCAACCAAAACAGAAAACT | TTACCCAATGTTCCTCCGGG | 110 | 95.44 |
| CYP337B5 | CCATCTGCGACAAAACCCTT | GGCTCCAGAGTCTTCTTCGT | 144 | 101.7 |
| CYP338A1 | AGCCACATTCCGACGTAGAA | TTCTCTTGCACCTCGGGATT | 130 | 100.7 |
| CYP340AA1 | TCATTGTGTCGTTCTGTGCG | GATGAGCGTGCCCCAATATC | 108 | 99.78 |
| CYP340AB1 | AACTACACACTGAGAGCGGG | AAAACGCATTGGGATTGGCT | 145 | 97.45 |
| CYP340K4 | GCACATTAGGTATTGAAGCCGT | TATGACGTCACTGAGGAGCC | 125 | 98.32 |
| CYP340L1 | TGCGAGAAACCTTCAAACCG | ACTCTCTTGCATCTGGTCCC | 122 | 99.70 |
| CYP341A11 | CCCTCCCGTGCCACTTATTA | GTCGGGGTCAAATTTCTCGG | 157 | 98.56 |
| CYP341B26 | GTGTGTGAAACTGCTCTCGG | ACCATACGCTCGGCTATCAA | 107 | 93.67 |
| CYP341B27 | AGCCTTCCTCGGTACACAAA | ACCCTGGGTCGATTTGTTCT | 114 | 94.45 |
| CYP354A14 | GCGAAACTGGCTATGGTTGA | TCTACACGAACCCACAGACC | 131 | 96.45 |
| CYP366A1 | CTGATGGTGTACAGGGGTGT | GCAGAACAGCAACGCATTTC | 100 | 98.65 |
| CYP367A1 | CAGCCAGTTGGTTGGTTTG | GAGTGGAAGTGCGGTTGGA | 86 | 99.07 |
| CYP367B1 | AAATTCTGGCTCGGTCCTGA | TTGTATTGCGGCCCTTTCAC | 104 | 98.79 |
| CYP9A10 | GGCGTTGCCCTTGACAGAC | ACGGTGGATTGGATAAACTGG | 126 | 97.67 |
| CYP9A27 | ATGACGATAAACCCAATG | CAAAGCCAGCGATAAAGA | 141 | 95.78 |
| CYP9A98 | CTACCAGCATCTGCGTCAC | TTAGCCTACACCTTAACCAAT | 122 | 94.09 |
| CYP9A11 | ATCTTACAACTCGCTACGC | GCTGCTGTCCTACCCATTA | 112 | 94.56 |
| CYP9A97 | TTTGCGCTTTGTGAGATAA | CTAAGTCCTTGCCCTGAAC | 159 | 93.78 |
| CYP9A9 | ACTACCCGCTACACAAACGA | CTCTTTCCCCAGTGCGTAGA | 102 | 92.89 |
| CYP339A1 | CAGATTTACGGTGGATTGT | CTTCATTGATGCCGCTTAT | 115 | 91.45 |
| CYP324A6 | GATGAACTCGTGGAAGCAG | ACCAGCCCATGATAGCGTA | 127 | 98.56 |
| CYP428A1 | AGCCATGAGACTCCATCCAG | TTTGCTTGTGACACCGTGAG | 124 | 104.23 |
| GAPDH  Actin | CTGAGGAACAGGTCGTGTCATC  AAGCCTTCGATGCCACCGGGTA | GATCGATAACGCGGTTGGAGTA  TTCGGGCGTGTTTAGTGGAGGC | 150  170 | 99.67  99.73 |
