# Supplementary material for: Changes in both trans- and cis-regulatory elements mediate insecticide resistance in a lepidopteron pest, Spodoptera exigua
Source: PLoS Genet. 2021 Mar 9;17(3):e1009403. doi: 10.1371/journal.pgen.1009403 (PMC7978377; doi:10.1371/journal.pgen.1009403)
Supplement: S3 Table — (DOCX) [file pgen.1009403.s003.docx]

**Table S3 Primers used in quantitative real-time PCR of transcription factors**

| **Gene** | **Forward primers** | **Reverse primers** | **Amplicon**  **(bp)** | **Efficiency**  **(%)** |
| --- | --- | --- | --- | --- |
| Maf | GTCGTCGCACTCTGAAGAAC | CCTGCATCAACTCCATGTCG | 120 | 98.49 |
| CncC | GGGACAGGAAACAGAGGACA | ATTGTTGGGAGGATAGCGGT | 144 | 98.78 |
| USP | TGAAGAGGGAGGCAGTTCAG | CGCTCGATTGACAACTCCTG | 97 | 98.56 |
| Dfd | CTCCAAGACCACCAGAACCT | CCCAAGGCATGCAGTTTTCT | 157 | 96.65 |
| BR-C | AAAGAACAACACCCACGCTC | GCTCGTTCTTCACATCGTCC | 113 | 99.02 |
| DorsalA | AAGTCAGAGCCAAGAGACCG | GTTCGTATTGAGGCGCGTAG | 82 | 97.72 |
| DorsalB | CGATACAGATGATGAGCGCG | TTCACTCTTGGCTACGGTCA | 187 | 99.31 |
| CrebA | CATTGACCCGGAGATGCAAG | GAGGGTGCGGATTCAACATC | 186 | 101.6 |
| CrebB | TGGTGGAAGAGAATGGCACT | CCTGGGTCGGTAATGTGAGT | 135 | 100.3 |
| ECR | AGTGTGTGGTGCCAGAAAAC | CACACTGCATAATGGGAGGC | 126 | 99.21 |
| KR | CACCCAGTTCTCCACCTTCT | TGGGGCGAAGGTATATTGCA | 149 | 100.2 |
| P53 | TTCTTGTATTCCCACCGCCT | CGCTCTCATGGTAGTGTTGC | 181 | 97.23 |
| GAPDH | CTGAGGAACAGGTCGTGTCATC | GATCGATAACGCGGTTGGAGTA | 150 | 99.67 |
| β-Actin | AAGCCTTCGATGCCACCGGGTA | TTCGGGCGTGTTTAGTGGAGGC | 170 | 99.73 |
