# Supplementary material for: Changes in both trans- and cis-regulatory elements mediate insecticide resistance in a lepidopteron pest, Spodoptera exigua
Source: PLoS Genet. 2021 Mar 9;17(3):e1009403. doi: 10.1371/journal.pgen.1009403 (PMC7978377; doi:10.1371/journal.pgen.1009403)
Supplement: S4 Table — (DOCX) [file pgen.1009403.s004.docx]

**Table S4 Primers used for gene amplification of transcription factors**

| **Primers** | **Primer sequences (5'-3')** | **Usage** |
| --- | --- | --- |
| Kr-h1-F | ATGGAGTCTTTGTTACCTATTAAA | Cloning complete fragment |
| Kr-h1-R | TTATGAGTCGGATGCGGCAGCTGG | Cloning complete fragment |
| P53-F | ATGGAGCATCCTGATATTGGATAT | Cloning complete fragment |
| P53-R | TTACTGTGACGGTGTTCTTGTATT | Cloning complete fragment |
| CrebA-F | ATGGAGGCTTACTTCGACATCTCG | Cloning complete fragment |
| CrebA-R | TCACTTGCGAGCGCCGCGCGCCAC | Cloning complete fragment |
| CrebB-F | ATGGATGGAATGGTGGAAGAGAAT | Cloning complete fragment |
| CrebB-R | TCATTCTGTCTTTTGTTGACAGTA | Cloning complete fragment |
| DorsalA-F | ATGGCGCGCCGCGACCAGCCCTAC | Cloning complete fragment |
| DorsalA-R | TTACTGCGGGATCGTGTTGTTGAG | Cloning complete fragment |
| DorsalB-F | ATGGCGCGCCGCGACCAGCCCTAC | Cloning complete fragment |
| DorsalB-R | TTAATTATTTTGAAATTTGCTGCC | Cloning complete fragment |
