# Supplementary material for: Changes in both trans- and cis-regulatory elements mediate insecticide resistance in a lepidopteron pest, Spodoptera exigua
Source: PLoS Genet. 2021 Mar 9;17(3):e1009403. doi: 10.1371/journal.pgen.1009403 (PMC7978377; doi:10.1371/journal.pgen.1009403)
Supplement: S5 Table — (DOCX) [file pgen.1009403.s005.docx]

**Table S5 Primers used for Constuction of transgenic *Drosophila* and eukaryotic expression**

| **Name** | **Primer sequences (5'-3')** | **Usage** |
| --- | --- | --- |
| CYP321A8-1F | ATGTTGTTTTTACCTTTGAG | Cloning complete fragment |
| CYP321A8-1488R | TTAGACATTTCTGGGAACTA |  |
| CYP321A8-Xhol-1F | TTCAGGCGGCCGCGGCTCGAGC  AAAATGTTGTTTTTACCTTTGAG | Constuction of transgenic *Drosophila* |
| CYP321A8-XbaI-1488R | CCTTCACAAAGATCCTCTAGA  TTAGACATTTCTGGGAACTA |  |
| CYP321A8-1F+EcoRI+Kozak | GGCGCCATGGATCCGGAATTCGC  CACCATGTTGTTTTTACCTTTGAG | Constuction of pFastBacHTA vector |
| CYP321A8-1488R+XhoI | CTTGGTACCGCATGCCTCGAG  TTAGACATTTCTGGGAACTA |  |
| HaCPR-1F+EcoRI+Kozak | GGCGCCATGGATCCGGAATTCGC  CACCATGTCAGACAGCGCACAGGA | Constuction of pFastBacHTA vector |
| HaCPR-2064R+XhoI | CTTGGTACCGCATGCCTCGA  GTTAACTCCATACATCTGCTG |  |
| CYP321A8-F | AAACAACCCCAAGACGCATG | Q-PCR |
| CYP321A8-R | CCAATGCCAAAGATAGCCCC |  |
| Rp49-F | ACTCAATGGATACTGCCCAAGA | Q-PCR |
| Rp49-R | CAAGGTGTCCCACTAATGCATA |  |
