# Supplementary material for: Changes in both trans- and cis-regulatory elements mediate insecticide resistance in a lepidopteron pest, Spodoptera exigua
Source: PLoS Genet. 2021 Mar 9;17(3):e1009403. doi: 10.1371/journal.pgen.1009403 (PMC7978377; doi:10.1371/journal.pgen.1009403)
Supplement: S6 Table — (DOCX) [file pgen.1009403.s006.docx]

**Table S6 Primers used for cloning 5’-flanking regions**

| **Primers** | **Primer sequences (5' - 3')** |
| --- | --- |
| PCYP321A8-1 | ACTGGAAGTCTCCTGATAGAACCTGCT |
| PCYP321A8-2 | ACCTCAAAAAGAGCACGCTGCGCTATG |
| PCYP321A8-3 | AATTTGCAGCTCAGAAACAGTATACTGT |
| PCYP321A8-4 | CAGCCGTTCTAATTTTGCTGAGTCATGA |
| PCYP321A8-5 | TACTGAATACGAGATAACTCTCTGAACC |
| PCYP321A8-6 | CACGACTGAAAGGTTAAGGTTAAGGTTG |
| PCYP321A8-7 | GATAAGAGTTATCTAACATCGACTAATG |
| PCYP321A8-8 | CAGCTTTACCTCATGGTGACAGGCAGAA |
| CYP321A8-DNA-F | GGTCAATGAATTGTGTAACATCATC |
| CYP321A8-DNA-R | GCCTACTGCTACTAAACTCAAAGGT |
| Adaptor Primer1 | GTAATACGACTCATCATAGGGC |
| Nested Adaptor Primer2 | ACTATAGGGCACGCGTGGT |
