# Supplementary material for: Changes in both trans- and cis-regulatory elements mediate insecticide resistance in a lepidopteron pest, Spodoptera exigua
Source: PLoS Genet. 2021 Mar 9;17(3):e1009403. doi: 10.1371/journal.pgen.1009403 (PMC7978377; doi:10.1371/journal.pgen.1009403)
Supplement: S7 Table — (DOCX) [file pgen.1009403.s007.docx]

###### **Table S7 Primers used for reporter and promoter constructs**

| Gene | Primer sequences (5'-3') |
| --- | --- |
| Maf-F | TCGAATTTAAAGCTTGGTACCATGCCTCATGATTTAAAGGA |
| Maf-R | TCGAACCGCGGGCCCTCTAGACTATGGCTGTATTTCCAATT |
| CncC-F | TCGAATTTAAAGCTTGGTACCATGTTTGAAGAGGAATTGGTTCTG |
| CncC-R | TCGAACCGCGGGCCCTCTAGATCACTGGTCGTAGCTCTTGGCTTT |
| 321A8-Full-F | GCGTGCTAGCCCGGGCTCGAGGGTCAATGAATTGTGTAACATCATC |
| 321A8-Full-R | CAGTACCGGAATGCCAAGCTTGTCTCTAATATTAATAAAAAATCAC |
| 321A8-T1-F | ATTTCTCTATCGATAGGTACCGGTCAATGAATTGTGTAACAT |
| 321A8-T1-R | AGAAACAGTATACTGCTCGAGCGTCCGGATGTAAACAAATGT |
| 321A8-T2-F | ATTTCTCTATCGATAGGTACCCTAACATTTGTTTACATCCGG |
| 321A8-T2-R | AGAAACAGTATACTGCTCGAGGTACTAGTTCTTCGTAAATCG |
| 321A8-T3-F | GCGTGCTAGCCCGGGCTCGAGCTGTCCGATTTACGAAGAACTAGT |
| 321A8-T3-R | CAGTACCGGAATGCCAAGCTTGTCTCTAATATTAATAAAAAATCAC |
| 321A8-T4-F | GCGTGCTAGCCCGGGCTCGAGCAGTATACTGTTTCTGGGCT |
| 321A8-T4-R | CAGTACCGGAATGCCAAGCTTGTCTCTAATATTAATAAAAAATCAC |
| 321A8-CncC-M1-F | ATACAAgactTTTttcGTTTAGAAAACAAGTTTTTTTTTACACA |
| 321A8-CncC-M1-R | TCTAAACgaaAAAagtcTTGTATGGGTAACATAACAAAAAAATG |
| 321A8-CncC-M2-F | AGTCAgactTCAttcAAATTAGAACGGCTGTCTCGCAAAAAT |
| 321A8-CncC-M2-R | TAATTTgaaTGAagtcTGACTTTGGTTAACAAATAAAAACTT |
| 321A8F(-1941) | GCGTGCTAGCCCGGGCTCGAGGGTCAATGAATTGTGTAACATCATC |
| 321A8F(-1652) | GCGTGCTAGCCCGGGCTCGAGATCGGCAAACGAGCAGACGGATTAC |
| 321A8F(-1361) | GCGTGCTAGCCCGGGCTCGAGACACAACGCTGTGGTTATTTCACGC |
| 321A8F(-888) | GCGTGCTAGCCCGGGCTCGAGCCGCTGACATTTATCAAAAATATCT |
| 321A8F(-522) | GCGTGCTAGCCCGGGCTCGAGCCTAATCCCTAAAAGGTCGGCAACG |
| 321A8F(-142) | GCGTGCTAGCCCGGGCTCGAGCAGTATACTGTTTCTGGGCT |
| 321A8F(-446)R | GCGTGCTAGCCCGGGCTCGAGTTACCATCAGGTAACCAGTCTGCT |
| 321A8F(-446)S | GCGTGCTAGCCCGGGCTCGAGTTACCACCAGGTAATCCATCTGCT |
| 321A8F(-385) | GCGTGCTAGCCCGGGCTCGAGCTGTCCGATTTACGAAGAACTAGT |
| 321A8R(-1941) | CAGTACCGGAATGCCAAGCTTGTCTCTAATATTAATAAAAAATCAC |
| 321A8(-385)-M1F | GTGTGTAGTGCGCATTACTTATGTGTTTTGAGAAAAGAAAAC |
| 321A8(-385)-M1R | ATAAGTAATGCGCACTACACACTAAAGTAAGAGCCGAGATTC |
| 321A8(-385)-M2F | AAACAATATTCTGTTTTCCACGTGCTGGGTTTTTGCAAAAG |
| 321A8(-385)-M2R | GTGGAAAACAGAATATTGTTTTCTTTTCTCAAAACACATAA |
| 321A8(-385)-M3F | CACGTGCTGGATTTTTGCAAAAGGACACTGATAAATATATA |
| 321A8(-385)-M3R | TTTGCAAAAATCCAGCACGTGGAAAACACAATATTGTTTTC |
| 321A8(-385)-M4F | GGACACAGATTAAAATATAGTTTAGCTAAATAATAATGA |
| 321A8(-385)-M4R | TATATTTTAATCTGTGTCCTTTTGCAAAAACCCAGCACG |
| 321A8(-385)-M5F | ACTCAGCAAAGTTAGAACGGCTGTCTCGCAAAAATAGAGAT |
| Knirps-1F-pIB | TCGAATTTAAAGCTTGGTACCATGGACCAAAAGTGCAAAGTATGT |
| Knirps-1122R-pIB | TCGAACCGCGGGCCCTCTAGATCAGACTTTGGTGGTGAGGTCCAG |
