# Supplementary figures and images for: Changes in both trans- and cis-regulatory elements mediate insecticide resistance in a lepidopteron pest, Spodoptera exigua
Source: PLoS Genet. 2021 Mar 9;17(3):e1009403. doi: 10.1371/journal.pgen.1009403 (PMC7978377; doi:10.1371/journal.pgen.1009403)

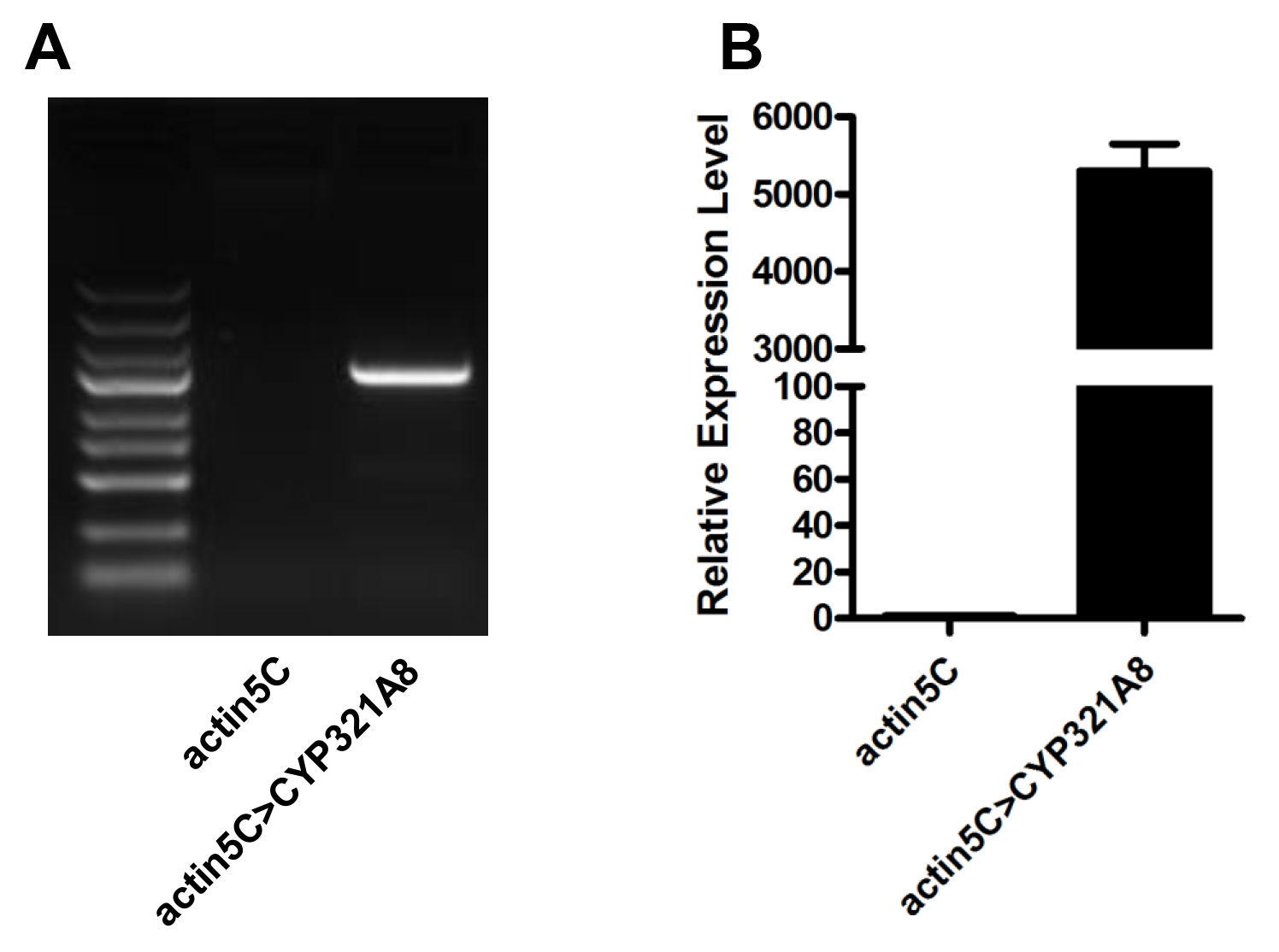

Supplement: S1 Fig — PCR was performed using the synthesized cDNA as a template and primers specific to CYP321A8 (A). In addition, the relative expression levels of the CYP321A8 transgene were assessed by qRT-PCR in the F1 progeny under the Act5C driver (B). The data shown are the mean ± standard error of the mean (n = 3). (TIF) [file pgen.1009403.s008.tif]

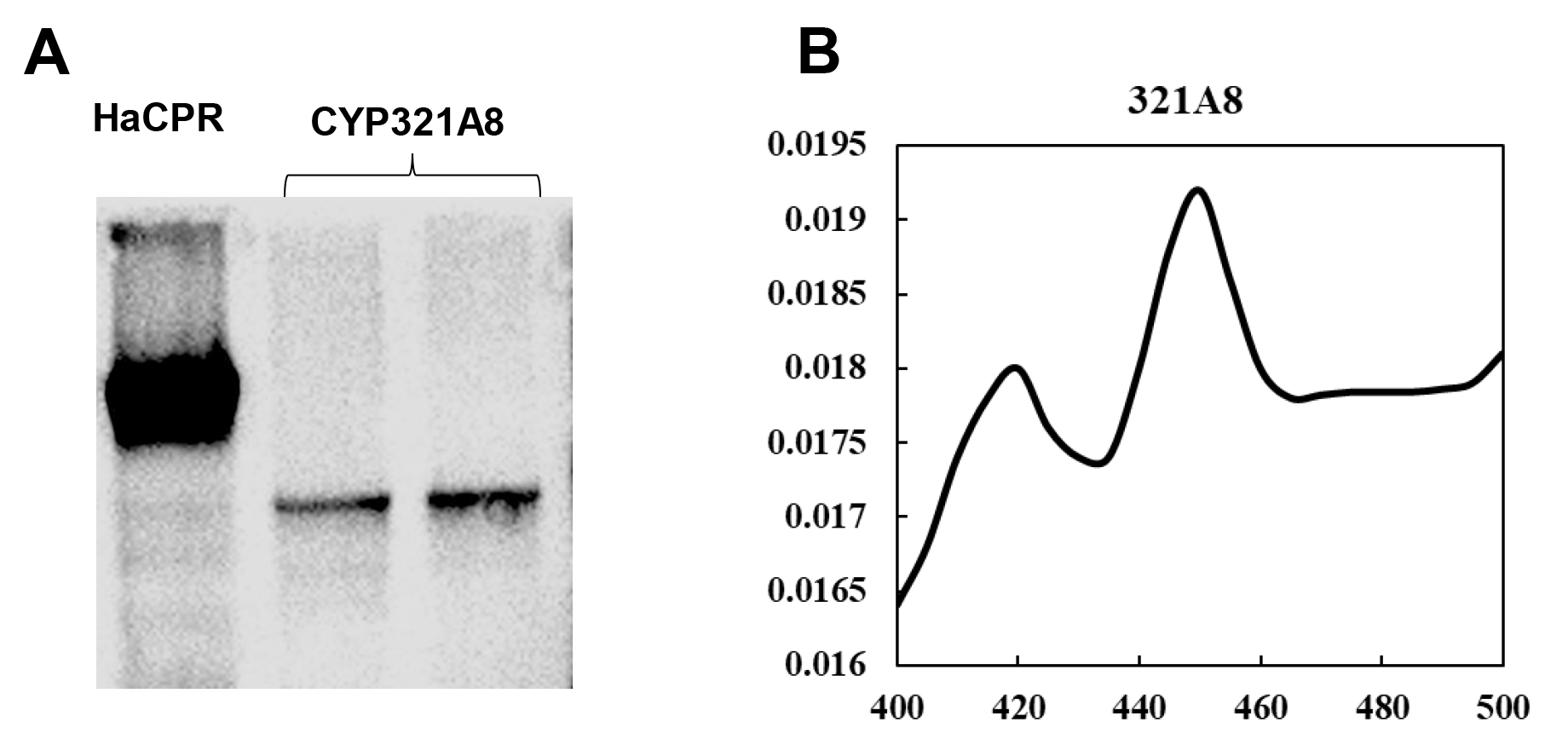

Supplement: S2 Fig — A) Western blot analysis of CYP321A8 expression in microsomes prepared from baculovirus-infected Sf9 insect cells. (B) Reduced CO-difference spectrum of the recombinant CYP321A8. (TIF) [file pgen.1009403.s009.tif]

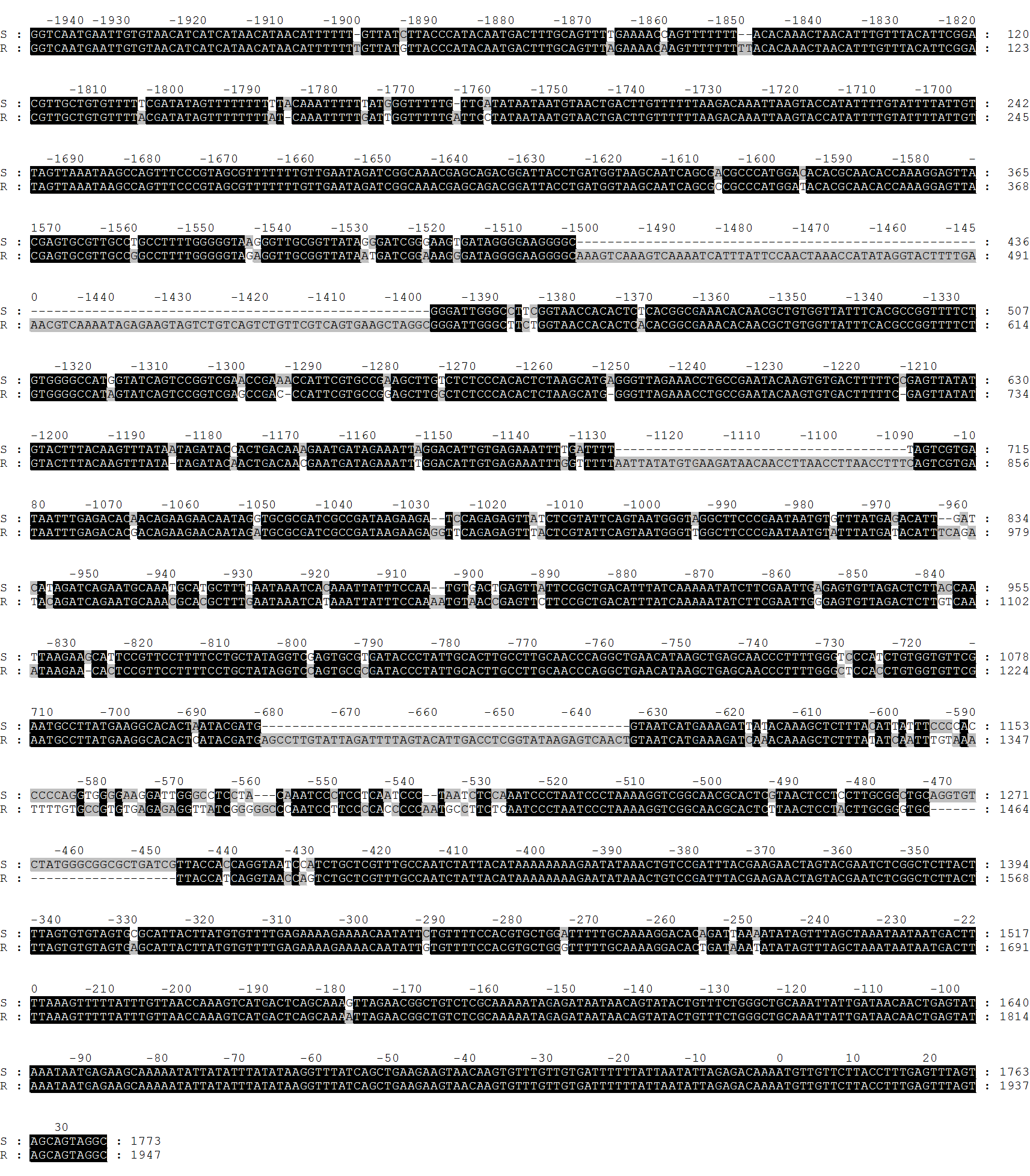

Supplement: S3 Fig — The nucleotides are numbered relative to the translation start site (ATG), with sequence upstream of it preceded by “-“. (TIF) [file pgen.1009403.s010.tif]
